# A pH-responsive T_1_-T_2_ dual-modal MRI contrast agent for cancer imaging

**DOI:** 10.1038/s41467-022-35655-x

**Published:** 2022-12-26

**Authors:** Hongwei Lu, An Chen, Xindan Zhang, Zixiang Wei, Rong Cao, Yi Zhu, Jingxiong Lu, Zhongling Wang, Leilei Tian

**Affiliations:** 1 Department of Materials Science and Engineering, Southern University of Science and Technology, Shenzhen, Guangdong 518055 China; 2 Department of Radiology, Shanghai General Hospital, School of Medicine, Shanghai Jiaotong University, Shanghai, 200080 China

**Keywords:** Imaging techniques and agents, Cancer imaging, Cancer imaging

## Abstract

Magnetic resonance imaging (MRI) is a non-invasive imaging technology to diagnose health conditions, showing the weakness of low sensitivity. Herein, we synthesize a contrast agent, SPIO@SiO_2_@MnO_2_, which shows decreased T_1_ and T_2_ contrast intensity in normal physiological conditions. In the acid environment of tumor or inflamed tissue, the manganese dioxide (MnO_2_) layer decomposes into magnetically active Mn^2+^ (T_1_-weighted), and the T_1_ and T_2_ signals are sequentially recovered. In addition, both constrast quenching-activation degrees of T_1_ and T_2_ images can be accurately regulated by the silicon dioxide (SiO_2_) intermediate layer between superparamagnetic iron oxide (SPIO) and MnO_2_. Through the “dual-contrast enhanced subtraction” imaging processing technique, the contrast sensitivity of this MRI contrast agent is enhanced to a 12.3-time difference between diseased and normal tissue. Consequently, SPIO@SiO_2_@MnO_2_ is successfully applied to trace the tiny liver metastases of approximately 0.5 mm and monitor tissue inflammation.

## Abstract (web summary)

Magnetic resonance imaging (MRI) is a well-established non-invasive medical imaging technology. Here, to improve the performance of the technique, the authors describe the design of a pH-responsive T1-T2 dual-modal MRI contrast agent, showing enhanced imaging sensitivity in preclinical cancer models.

## Introduction

Magnetic resonance imaging (MRI) is the most powerful technique for noninvasive tomographic imaging of biological targets for its high spatial resolution, unlimited penetration depth, and soft tissue discrimination ability^1–5^. However, MRI still suffers from its low inherent sensitivity, which limits the applications for diagnosis at an early stage of disease and small lesions^6–10^. In MRI, the relaxation time of water protons is measured, and contrast agents can alter locoregional magnetic fields and accelerate proton relaxation processes, subsequently enhancing the MRI sensitivity and diagnostic accuracy. Paramagnetic materials, which facilitate the spin-lattice relaxation of protons causing a positive MRI, are called T_1_ contrast agents. T_2_ contrast agents, mainly referred to as iron oxide (SPIO), cause protons in their vicinity to undergo spin-spin relaxation, which displays negative contrast on T_2_-weighted MRI images. Compared with single-mode contrast agents^11–16^, recently, T_1_–T_2_ dual-modal MRI contrast agents can significantly improve diagnostic accuracy by providing paired anatomical images at the same levels but with different contrasts^17–22^. Moreover, the activatable MRI contrast agents that can recognize tumor microenvironments and change their contrasts, will also enhance the differences between normal and disease tissues^23^. Cheon and co-workers reported a magnetism-based nanoscale distance-dependent magnetic resonance tuning (MRET) system^24^. In MRET, the T_1_–weighted signal of a paramagnetic contrast agent will be quenched by a superparamagnetic T_2_ contrast agent at a critical distance and recovers when they separate apart. Such MRET systems could be designed sensitively in response to biological events, such as enzymolysis, pH variation, and protein-protein interaction, realizing sensitive detection in vivo.

It is no doubt that utilizing a T_1_ and T_2_ two-way activatable contrast agent for T_1_–T_2_ dual-modal MRI will result in doubly improved detection sensitivity. In our previous work^25^, we brought the T_1_ and T_2_ contrast agents together in the micelles and found both the signals on T_1_ and T_2_ weighted MRI images were significantly reduced. When the presence of GSH disassembled the micelle structure, the MRI signals on T_1_ and T_2_ weighted images were dually recovered as the T_1_ and T_2_ contrast agents dissociated apart. Therefore, both T_1_ and T_2_ contrast differences were produced between the diseased and normal tissues by responding to the GSH biomarker. Further, by a method called “dual-contrast-enhanced subtraction imaging (DESI)”, the T_1_ and T_2_ contrast differences can be overlaid to enhance the imaging sensitivity. This work inaugurates an era for T_1_–T_2_ dual-mode MRI detection.

**Fig. 1:**
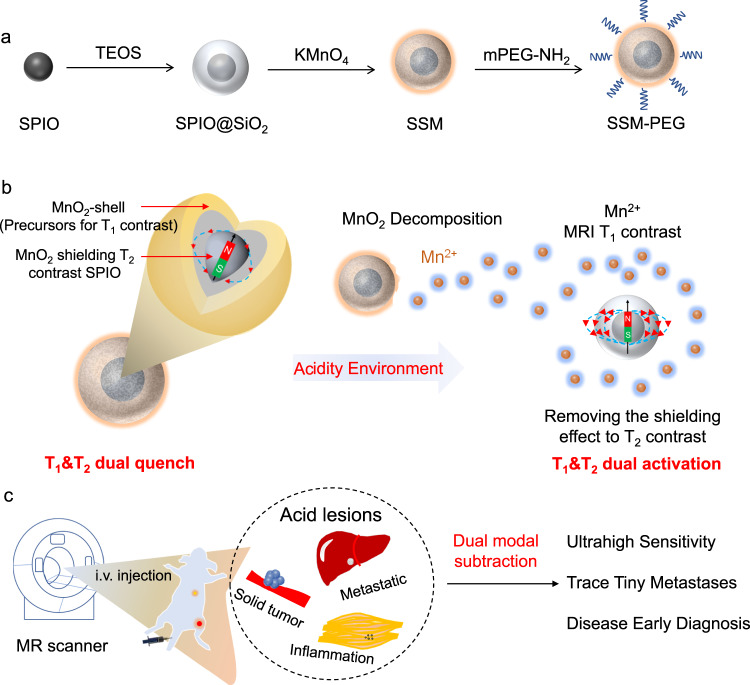
Schematic illustration of the structure, working mechanism, and applications of the pH-responsive T_1_ and T_2_ dual-modal contrast agent. **a** The synthesis and structure of the contrast agent SPIO@SiO_2_@MnO_2_ (SSM). SPIO represents superparamagnetic iron oxide and TEOS represents tetraethyl orthosilicate; **b** SPIO@SiO_2_@MnO_2_ shows weak T_1_ and T_2_ contrast intensity in normal physiological conditions, as the T_2_ signal of SPIO is quenched by the MnO_2_ layer. In the acid environment of tumor or inflamed tissue, the MnO_2_ layer will decompose into magnetically active Mn^2+^ (T_1_-weighted), and the T_1_ and T_2_ signals are sequentially recovered. **c** Using dual-contrast-enhanced subtraction imaging (DESI) strategy enhances the imaging quality for disease diagnosis.

In this work, a pH-activatable T_1_–T_2_ dual-modal MRI contrast agent, SPIO@SiO_2_@MnO_2_, is designed and synthesized. In the normal tissue, MnO_2_ acts as an inactive T_1_ contrast agent precursor, which can efficiently quench the T_2_ weighted signal of SPIO. In tumor or inflammation tissue with an acid environment, MnO_2_ decomposes into Mn^2+^ and sequentially increases both T_1_ and T_2_ weighted signals. The images are further processed by the DESI method;^25^ the biggest contrast difference of 12.3 times between diseased and normal tissue has been reached (Fig. 1). Different from the MRET with a critical distance, the quenching effect from MnO_2_ to the T_2_-weighted signal of SPIO is found reversely proportional to their separation distance, i.e., the closer the quenching effect becomes stronger. As a result, both contrast quenching-activation degrees of T_1_ and T_2_ images can be accurately tuned by regulating the distance between SPIO and MnO_2_ through the SiO_2_ intermediate layer. Finally, we demonstrate that SPIO@SiO_2_@MnO_2_ nanoprobe can sensitively offer high-quality image information by distance regulation and be applied to detect small lesions and identify the various acid environment.

## Results

### Dual-activatable mechanism of MRI T_1_ and T_2_ contrast signals

**Fig. 2:**
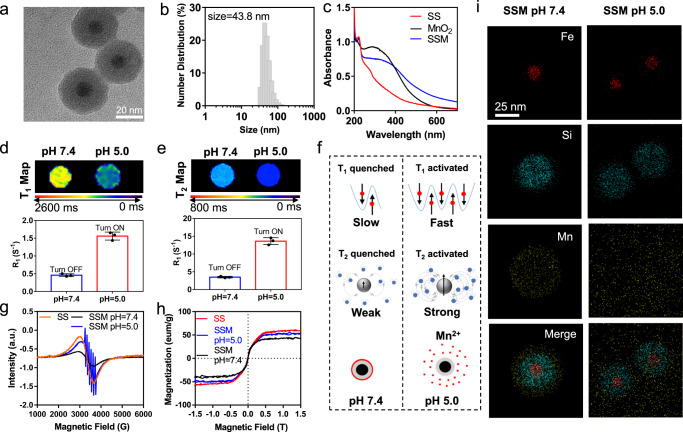
Characterization of SSM (SPIO@SiO_2_@MnO_2_) and the pH-activatable properties. **a** TEM micrograph and **b** size distribution of SSM. Representative data are shown from three independently repeated experiments. **c** Absorption spectra of SS (SPIO@SiO_2_), MnO_2_, and SSM. **d** T_1_ Map & R_1_, and **e** T_2_ Map & R_2_ of SSM, at pH 7.4 and 5.0 respectively. T_1_ and T_2_ Map are representative data from three independent samples. R_1_ and R_2_ data are presented as mean ± SD from three independent samples. **f** illustration of the dual pH-activatable mechanism. **g** EPR spectroscopic analysis of SS at pH 7.4 and SSM at pH 7.4 and 5.0. **h** VSM analysis of SS at pH 7.4 and SSM at pH 7.4 and 5.0. **i** EDX element mapping images of SSM at pH 7.4 and 5.0 for Fe, Si, and Mn. Representative data are shown from two independently repeated experiments. Source data are provided as a Source Data file.

In this study, the nanohybrids with core/shell/shell structures were synthesized, in which the T_2_ core was the superparamagnetic iron oxide (SPIO) nanoparticle. The first shell was a SiO_2_ layer, and a thin layer of MnO_2_ was deposited as the second shell. The final nanoparticle (SPIO@SiO_2_@MnO_2_) was named SSM, whose core–shell-shell structure was confirmed by TEM characterization (Fig. 2a). The size of the SPIO core was determined to be about 12.8 nm, and the thickness of SiO_2_ and the MnO_2_ shell were 12.4 nm and 2.2 nm. Consistently, the hydrodynamic diameter of SSM measured by DLS was 43.8 nm (Fig. 2b). The UV–vis spectrum of SSM showed a characteristic absorption peak at 302 nm (Fig. 2c), which could be assigned to the absorbance of MnO_2_^26^.

The magnetic properties of SSM were investigated. As shown in Supplementary Fig. 1, no resonance was observed in the electron paramagnetic resonance (EPR) spectrum of pure MnO_2_, illustrating the unpaired electrons in manganese atoms were “locked” in the oxidation state. Accordingly, SSM showed a quenched T_1_ signal. According to the vibrating sample magnetometer (VSM) test in Fig. 2h, SSM showed a marked reduction in magnetization in comparison with SPIO@SiO_2_ (SS), which demonstrated that MnO_2_ would shield the magnetic field of the SPIO core. Therefore, in addition to the quenched T_1_ weighted signal, SSM also showed a quenched T_2_ weighted signal. The dual “signal quenching” property of SSM would promise a low background in imaging applications.

For the quenching and activation of the T_1_ signal, it is well known that the MnO_2_ layer is pH -sensitive^27^, which will decompose into magnetically active Mn^2+^ at the acid condition and significantly change the MRI signal of SSM. Thus, the pH sensitivity of SSM nanoparticles was investigated by measuring the MRI signals in buffers with pH values of 7.4 and 5.0, respectively. From the MRI map images in Fig. 2d, e, both T_1_ and T_2_ (T_1_ = Longitudinal relaxation time; T_2_ = Transverse relaxation time) were decreased as the pH value changed from 7.4 to 5.0. Both R_1_ and R_2_ values (*R*_1_ = 1/T_1_, *R*_2_ = 1/T_2_) were low at pH 7.4 and raised at pH 5.0. Thus, these results indicated that SSM showed dual-activatable T_1_ and T_2_ contrasts in response to the acid environment, which was believed to be the result of the decomposition of the MnO_2_ outer layer. In the electron paramagnetic resonance (EPR) measurements of Fig. 2g, SSM at a pH value of 7.4 and SS showed similar doublet resonances at around 2950 and 3650 Gauss, which were the characteristic resonances of SPIO. Whereas after incubation at pH 5.0, SSM exhibited the typical peaks of Mn^2+^ in addition to the peaks of SPIO. Those EPR results suggest that MnO_2_ on the surface of SSM decomposed and released Mn^2+^ under the acid condition, which was visibly confirmed by the EDS mapping images of SSM at pH 7.4 and 5.0 (Fig. 2i). The decomposition of the MnO_2_ shell led to two sequential results: the magnetically active Mn^2+^ was released and turned on the T_1_ weighted signal (the interaction between Mn^2+^ and surrounding water will shorten the longitudinal relaxation times of water protons), moreover, which further eliminated the MnO_2_ shield to the magnetic field of the SPIO core and resulted in “recovered” T_2_ contrast signal. According to the VSM analysis (Fig. 2h), SSM in its intrinsic state showed reduced magnetic saturation values, which corresponded to the quenched T_2_ contrast signal of SSM; After being incubated at pH 5.0, SSM uncovered its MnO_2_ shell, which recovered the magnetic field of the SPIO core to the original state (identified by SS). These results could well explain the pH-activatable T_2_ contrast signal, which is the sequential result of the turn-on of the T_1_ contrast signal. The dual activation mechanism was summarized in Fig. 2f, which determined that SSM would show high sensitivity in discriminating the diseased (with the acid environment) and normal tissues (with the neutral environment).

### The mechanism of the quenching effect on T_2_ contrast signal

**Fig. 3:**
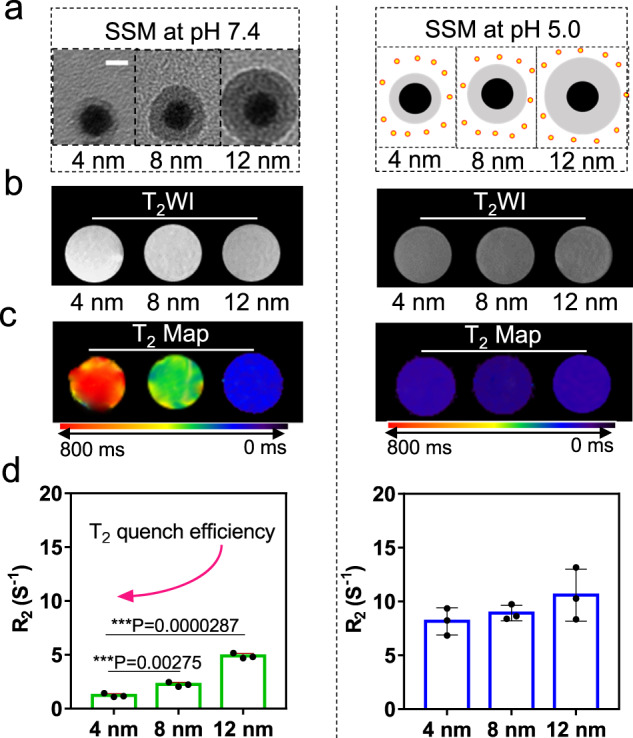
The distance-dependent quenching effect of MnO_2_ to T_2_ contrast signal of the SPIO core. **a** TEM images and schematic illustration of the structure of SSM (SPIO@SiO_2_@MnO_2_) with different SiO_2_ thicknesses (4, 8, and 12 nm) at pH 7.4 and 5.0, scale bar = 12 nm. The SiO_2_ intermediate layer works as a ruler to investigate the influence of distance on the T_2_ quenching effect; **b** T_2_-weighted images, **c** T_2_ Map, and **d** *R*_2_ of SSM samples with different SiO_2_ thicknesses (4, 8, and 12 nm) at pH 7.4 and 5.0. T_2_-weighted images and T_2_ Map are representative data from three independent samples. *R*_2_ data are presented as mean ± SD from three independent samples. The two-tailed Student’s *t*-test is employed for the statistical analysis. *P < 0.05; **P < 0.01; ***P < 0.001. Source data are provided as a Source Data file.

According to the above investigations on the SSM contrast agent, we observed that the T_1_ contrast signal of Mn^2+^ was “masked” by the formation of MnO_2_. Moreover, the MnO_2_ would quench the T_2_ contrast signal of the SPIO subsequently, resulting in the “dually (T_1_ and T_2_) quenched” state of the contrast agent in the neutral condition. Like one key for two locks, the decomposition of MnO_2_ could efficiently convert the contrast agent into the “dually (T_1_ and T_2_) activated” state in the acid environment. It has been clearly proved that the T_1_ contrast signal of a T_1_ contrast agent (paramagnetic properties) will be quenched by a T_2_ contrast agent (superparamagnetic properties) when their distance becomes close enough^24^. Therefore, we further tuned the distance between the SPIO core and the MnO_2_ layer by varying the thickness of the intermediate SiO_2_ layer. The thickness of the silica shell can be adjusted by controlling the reaction time (Supplementary Fig. 2). As a consequence, different SSM nanoparticles were synthesized with different SiO_2_ thicknesses (4, 8, and 12 nm) but the same amounts of SPIO and MnO_2_, which had been verified via ICP-MS analysis (Supplementary Table 1). Figure 3a presents the TEM image of these SSM nanoparticles and the schematic illustration of the decomposition of the MnO_2_ layer in the acid environment. We evaluated the MRI performance of the three SSM nanoparticles in the neutral or acid environment. As seen from Supplementary Fig. 3, without the coating of the MnO_2_ layer, SS with different SiO_2_ thicknesses showed identical R_1_ values. The R_2_ value of SS nanoparticles was reduced in comparison with naked SPIO. However, the R_2_ values of SS nanoparticles with various thicknesses of the SiO_2_ layer (4, 8, and 12 nm) showed similar intensity. The results revealed that the SiO_2_ layer mainly influenced the surface properties. After coating the MnO_2_ layer, the T_1_ signal showed no significant change with the SiO_2_ thickness decreasing (Supplementary Fig. 4), which was reasonable as MnO_2_ showed no magnetic activity. While the T_2_ weighted images of SSM dramatically changed from dark gray to bright (Fig. 3b), and the T_2_ signal was significantly decreased with decreasing the SiO_2_ thickness, as observed from the Map image in Fig. 3c and R_2_ value in Fig. 3d. All these results proved that the MnO_2_ layer would shield the magnetic field of the SPIO core, and the shielding effect became more significant as the MnO_2_ layer approached the SPIO core.

To reveal the mechanism of T_2_ signal quenching, we analyzed the T_2_ weighted signal of the SPIO with different diamagnetic shells. We found that at the same SiO_2_ shell thickness (4 nm), SS nanoparticles showed mild T_2_ signal quenching. When further coating Ag_2_O and MnO_2_, the nanoparticles showed enhanced T_2_ signal quenching (Supplementary Fig. 5). Therefore, the diamagnetic nature of the MnO_2_ or Ag_2_O layer would shield the magnetic field of the SPIO core and quench the T_2_-weighted signal to a certain degree. Similar phenomena have also been reported in other research works^28–30^. However, we also observed that the MnO_2_ layer shows a more significant quenching effect compared with Ag_2_O and SiO_2_. For the Ag_2_O diamagnetic shell, as the distance between SPIO and Ag_2_O increased, almost no distance effect could be observed (Supplementary Fig. 6), which was quite different from the result of the MnO_2_ diamagnetic shell. Moreover, in addition to the distance effect, we further measured the T_2_ weighted image of SPIO@SiO_2_@MnO_2_ at the same SiO_2_-shell thickness while changing MnO_2_ contents (Supplementary Fig. 7). The result indicated the T_2_ quenching effect was notably enhanced by changing Fe/Mn weight ratios from 1:1 to 1:40 while controlling the same SPIO amount and SiO_2_ thickness. The difference between MnO_2_ and Ag_2_O demonstrated that additional reasons (rather than the shielding effect of the diamagnetic shell) caused the MnO_2_ layer to quench the T_2_ signal of SPIO. We assumed the T_2_ quenching effect also came from the negative exchange interaction between Mn and Fe, which results in a non-collinear antiferromagnetic configuration in their combination^31^. Such exchange interactions were also observed in bi-magnetic core/shell nanoparticles, which would generate a large exchange bias as the two magnetic materials were coupled together. Moreover, it was reported that such magnetic exchange interaction did show distance dependence^32^.

We observed that, with a SiO_2_ interlayer of 4 nm, the R_2_ value of SSM showed a ~12.0 times decrease in comparison with SS (Supplementary Fig. 3); as a result, the T_2_ contrast signal of SSM was almost completely quenched. In comparison, SSM and SS showed a 6.6-time difference for 8 nm SiO_2_ interlayer and a 3.0-time difference for 12 nm SiO_2_ interlayer in *R*_2_ values (Fig. 3d left). After the decomposition of the MnO_2_ shell in an acid environment (pH=5.0), the R_2_ values of all three nanoparticles recovered to the same level (Fig. 3d right). At pH 7.4, the transverse relaxivity *r*_2_ of SSM with various SiO_2_ thicknesses of 4, 8, and 12 nm were 12.08, 21.06, and 35.38 mM^−1^ s^−1^, respectively. On the contrary, *r*_2_ values of SSM at pH 5.0 showed no notable differences (Supplementary Fig. 8). These results suggested that the shielding effect of the MnO_2_ layer on the SPIO core was distance-dependent. Therefore, the contrast sensitivity of the SSM probe can be accurately tuned by regulating the thickness of the SiO_2_ intermediate layer. The SSM nanoparticle with a 4 nm SiO_2_ layer was used for all the following experiments. The longitudinal relaxivity (*r*_1_, 1.34 and 4.60 mM^−1^ s^−1^) and transverse relaxivity (r_2_, 13.88 and 82.21 mM^−1^ s^−1^) of SSM at pH value of 7.4 and 5.0 were displayed in Supplementary Fig. 9, the *r*_1_ and *r*_2_ of SSM both enhanced due to the environment pH changed from 7.4 to 5.0.

### MRI discriminates between the normal and cancer cells

**Fig. 4:**
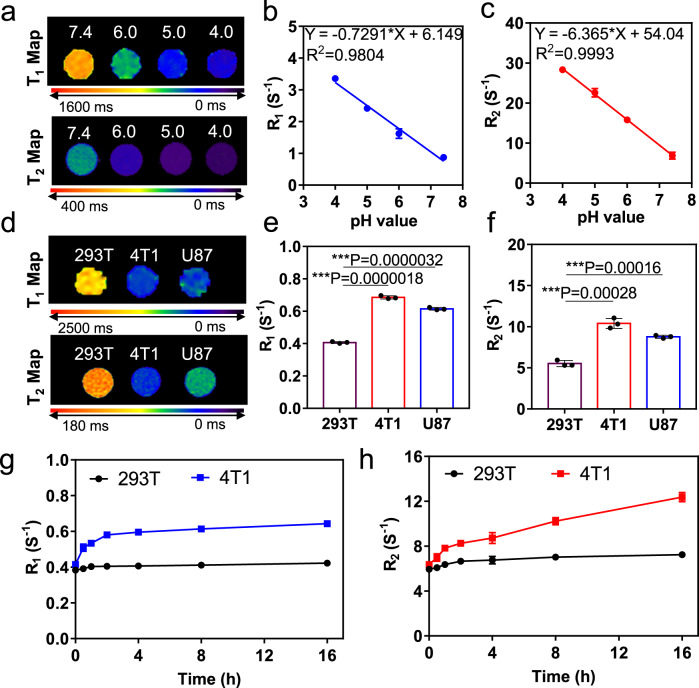
Capability of SSM (SPIO@SiO_2_@MnO_2_) in pH discrimination between normal and cancer cells. **a** T_1_ Map and T_2_ Map of SSM in different pH environments (pH = 7.4, 6.0, 5.0, and 4.0). Representative data are shown from three independent samples. **b** R_1_ and **c** R_2_ of SSM in different pH environments (pH = 7.4, 6.0, 5.0, and 4.0). Data are presented as mean ± SD from three independent samples. **d** T_1_ Map and T_2_ Map of different cell lines (293T, 4T1, and U87) incubated with SSM. Representative data are shown from three independent samples. **e** *R*_1_ and **f** *R*_2_ of different cell lines (293T, 4T1, and U87) incubated with SSM. Data are presented as mean ± SD from three independent samples. **g** *R*_1_ and **h** *R*_2_ of 293T and 4T1 cell lines incubated with SSM for different periods (0.5, 1, 2, 4, 8, and 16 h). Data are presented as mean ± SD from three independent samples. The two-tailed Student’s *t*-test is employed for the statistical analysis. *P < 0.05; **P < 0.01; ***P < 0.001. Source data are provided as a Source Data file.

The pH-dependent MRI property of SSM nanoparticles was further investigated. As shown in Supplementary Fig. 10, in the pH range from 7.4 to 4.0, the T_1_ weighted images showed gradually “activated” signals (becoming brighter and brighter); meanwhile, the T_2_ weighted images were also “activated” (becoming darker and darker). Calculated from the MRI map images in Fig. 4a, both *R*_1_ and *R*_2_ values were decreased as the pH value decreased. For the excellent sensitivity of SSM in pH detection, it is worth noting that there was a good linear relationship between the *R*_1_/*R*_2_ values and the pH values (Fig. 4b, c). Also, in addition to the pH-dependent properties, both the T_1_ weighted image, T_1_ map image, T_2_ weighted image, and T_2_ map image of SSM showed a time-dependent response to the acid environment (Supplementary Fig. 11). As SSM could sensitively indicate the change of less than 1.0 pH unit, we tried to utilize SSM as the MRI probe to discriminate between the normal and cancer cells. Generally, cancer cells showed higher glycolytic activity than normal cells; therefore, cancer cells would uptake and produce more lactate acid, resulting in acidic intracellular and extracellular environments^33^. According to the previous investigations, the pH measured at the surface of the highly metastatic cells was pH 6.7–6.8, and within tumors was around pH 6.1–6.4^34^. As a result, metastatic cells can be 0.6–1.3 pH units lower than normal cells (pH 7.4). Three different cell lines, 293T, 4T1, and U87, were selected and incubated with SSM for 4 h. 293T is a normal cell line, while both 4T1 and U87 are highly metastatic cancer cells. From the data in Fig. 4d–f and Supplementary Fig. 12, both the T_1_ and T_2_ images in the 293 T group were kept low contrast signals; on the contrary, the corresponding images in the two tumor cell groups (4T1 and U87) were all activated to high signal levels. The time dependency of dual-active MRI signals incubated in 4T1 cells and 293T cells were also investigated. As shown in Supplementary Figs. 13, 14 and Fig. 4g, h, both the *R*_1_ and *R*_2_ values of the 4T1 cell group exhibited a time-dependent increasing process, whereas the 293T cell group did not show obvious changes in *R*_1_ and *R*_2_ values. As the cancer cells showed a more acidic intracellular environment, SSM nanoparticles showed gradually activated signals on T_1_ and T_2_ images along with the intracellular uptake of the SSM nanoparticles. These results proved that SSM was an MRI probe that can sensitively respond to pH changes, showing great potential in tumor and inflammation diagnosis in vivo.

### MRI detection on the in vivo xenograft model

For in vivo application, PEG_5K_-NH_2_ modified SSM (SSM-PEG) was synthesized to enhance the biostability and biocompatibility of the MRI probe. The PEG attached to the SSM surface was due to the Mn-N coordinate bonding between PEG-NH_2_ and MnO_2_. The size and zeta potential results (Supplementary Figs. 15 and 16) displayed that SSM-PEG showed a larger size and more neutral surface potential in comparison with SSM due to the surface modification of PEG. Further, the T_1_ and T_2_ map images of SSM and SSM-PEG in different pH buffers (pH=7.4, 6.0, and 5.0) were tested and compared. The calculated R_1_ and R_2_ values were summarized in Supplementary Fig. 17, demonstrating that the PEGylation modification would not affect the pH-activation process. In SSM-PEG, the SSM part could still be freely accessible to the environment as the PEG shell was hydrophilic and loosely packed. The stability test in Supplementary Fig. 18 indicated that SSM-PEG could keep stable for nearly 48 h in a 10% Fetal Bovine Serum (FBS) solution. It should be noted that the DLS analysis was conducted by the “intensity weighted distribution” method here to eliminate the interferences from a large number of FBS molecules. However, the “intensity weighted distribution” method will be more biased to the larger nanoparticles than the previously used “number weighted distribution” method; therefore, a relatively larger size of SSM-PEG was displayed. In addition, cytotoxicity tests were carried out to evaluate the safety of SSM-PEG. Both SSM and SSM-PEG exhibited no significant cytotoxicity in the 4T1 cell line after 24 h incubation (Supplementary Fig. 19). Moreover, the cytotoxicity of Mn^2+^ was investigated (Supplementary Fig. 20). Mn^2+^ with a concentration up to 1000 μM still showed negligible cytotoxicity (>85% viability). Therefore, considering the dosages we used, no obvious toxicity would be induced by Mn^2+^ release from the SSM nanoprobe. What’s more, the Prussian blue staining experiment in Supplementary Fig. 21 proved that SSM-PEG showed a good cell uptake property. The hemolysis assay is a universal method to evaluate the hemocompatibility of materials. Thus, hemolysis study was carried out to further assess the biocompatibility of SSM-PEG. The results (Supplementary Fig. 22) proved that SSM-PEG displayed a low cytotoxic response at two different concentrations of 50 μg/mL and 100 μg/mL (<5% of hemolysis); on the contrary, SSM without PEGylation under the same concentrations showed marked hemolysis (>50% of hemolysis). The results demonstrated that the PEGylation was important for improving the biocompatibility of the SSM probe, making it safe for intravenous administration. The pharmacokinetics studies on SSM-PEG and SSM further proved the critical role of PEGylation in in vivo application. The blood circulation time *t*_1/2_ of SSM-PEG increased to nearly 82.8 min (Supplementary Fig. 23), while t_1/2_ of SSM was 29.0 min. The PEG stealth layer will protect the particle from uptake by the reticuloendothelial system and elongate the blood circulation time.

**Fig. 5:**
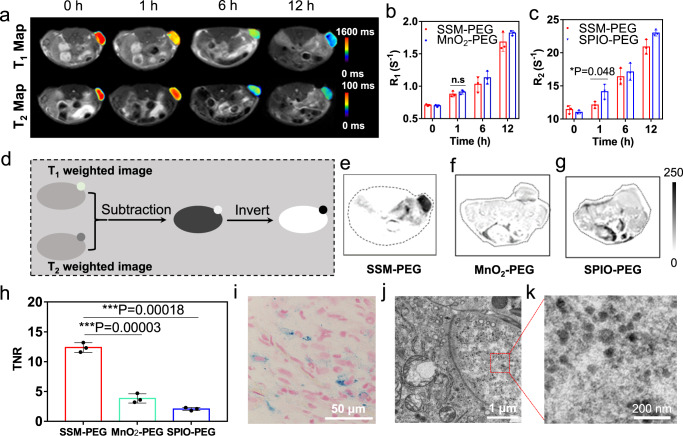
SSM-PEG (PEG-modified SPIO@SiO_2_@MnO_2_) for in vivo MRI of 4T1 xenograft tumor-bearing mice. **a** T_1_ and T_2_ Map, **b** *R*_1_, and **c** *R*_2_ at the tumor site of the 4T1-bearing mice. The I.V. injections of SSM-PEG and MnO_2_-PEG were given a dosage of 5 mg/kg, and the MRI diagnosis was performed at different time points at 0, 1, 6, and 12 h post the injection. T_1_ and T_2_ Map are representative data from three mice samples. *R*_1_ and *R*_2_ Data are presented as mean ± SD from three independent mice samples. **d** Illustration of the Dual-contrast-Enhanced Subtraction Imaging (DESI) procedures; T_1_ and T_2_ dual-modal MRI images were processed by DESI method of the tumor-bearing mice at 12 h after the injection of **e** SSM-PEG, f MnO_2_-PEG, and **g** SPIO-PEG. Representative data are shown from three mice samples. **h** The tumor-to-normal tissue signal ratios (TNRs) quantified from the DESI processed images in **e**–**g**. Data are presented as mean ± SD from three independent mice samples. **i** Prussian blue stain and (**j**) and (**k**) TEM images of the tumor slices from the mice receiving the injection of SSM-PEG. Representative data are shown from three mice samples. The two-tailed Student’s t-test is employed for the statistical analysis. *P < 0.05; **P < 0.01; ***P < 0.001. Source data are provided as a Source Data file.

Based on the good biostability and biosafety of SSM-PEG, we further investigated the feasibility of SSM-PEG as the MRI contrast agent in vivo. First, SPIO-PEG and MnO_2_-PEG were prepared as control materials to test whether SSM-PEG would well go through pH-activation in vivo in the tumor acidic environment. SPIO-PEG was synthesized in the absence of the MnO_2_ layer, and MnO_2_-PEG was synthesized without the SPIO@SiO_2_ core. SSM-PEG, MnO_2_-PEG (the control group of pH-activatable T_1_ contrast image), and SPIO-PEG (the control group of T_2_ contrast image after activation) were intravenously (I.V.) injected into the 4T1 breast subcutaneous tumor model and performed the MRI measurement. After injection, the tumor sites of the mice treated with SSM-PEG showed gradually enhanced both T_1_ and T_2_ contrast in the MR images, along with a time increase (Supplementary Fig. 24). While the mice treated with MnO_2_-PEG (Supplementary Fig. 25) only showed enhanced T_1_ contrast in tumor image, and SPIO-PEG (Supplementary Fig. 26) only showed enhanced T_2_ contrast. As shown in Fig. 5a–c, The T_1_ contrast signals at the tumor sites were all similar for the MnO_2_-PEG and SSM-PEG groups at different time points post-injection. In contrast, SSM-PEG displayed a visibly lower T_2_ contrast in comparison with SPIO-PEG at the first hour; after that, SSM-PEG and SPIO-PEG reached a similar level of MRI T_2_ contrast signal at 12 h post injection. Such a phenomenon accorded with the fact that SSM-PEG needed to go through the activation process to give the MRI signals. At 12 h after injection, both the T_1_ and T_2_ contrast signal of the SSM-PEG group in the tumor site approached the levels same as that of SSM-PEG and SPIO-PEG, respectively, indicating the full activation of the SSM-PEG probe. Due to the T_1_&T_2_ dual-modal properties of SSM-PEG after the activation in the acid-tumor environment, we could further use the dual-contrast-enhanced subtraction imaging (DESI) method to sum up the dual contrast and subsequently improve the diagnosis resolution. As shown in Fig. 5d, the DESI was carried out by subtracting the T2-weighted image from the T_1_ weighted image and further inverting the resultant image. As shown in Fig. 5e–g (The details of the subtraction process and origin images were displayed in Supplementary Fig. 27), compared with MnO_2_-PEG and SPIO-PEG that are single-modal (T_1_ or T_2_) contrast agents, the dual-modal activatable SSM-PEG showed a significantly enhanced imaging sensitivity after the DESI treatment, the tumor site displayed remarkable dark gray MRI signal while the normal tissue showed nearly “zero” background in the SSM-PEG group. Further, the tumor-to-normal tissue signal ratios (TNRs) of the three contrast agents were quantified from their T_1_-weighted images, T_2_-weighted images, and DESI-processed images. We observed that SSM-PEG showed a similar T_1_ weighted TNR and a relatively higher T_2_ weighted TNR in comparison with MnO_2_-PEG; meanwhile, SSM-PEG showed a similar T_2_ weighted TNR and a relatively higher T_1_ weighted TNR in comparison with SPIO-PEG. Therefore, as SSM-PEG could improve both the T1-weighted TNR and the T2-weighted TNR, the DESI processing method can significantly improve the TNR value of the SSM-PEG group. In contrast, DESI processing showed no visible enhancing effect on the TNR value of the SPIO-PEG (in comparison with its T_2_ weighted TNR) and MnO_2_-PEG groups (in comparison with its T_1_ weighted TNR). From Fig. 5h, the TNR value for SSM-PEG quantified from the DESI processed images was 12.3 times; on the contrary, the TNR values for MnO_2_-PEG and SPIO-PEG were 3.8 and 2.1, respectively. Thus, we demonstrated that by using the T_1_&T_2_ dual-activatable SSM probe and processing the MRI images through the DESI method, the resolution of in vivo MRI images could be remarkably improved. Furthermore, the Prussian blue stain (to indicate the ferric iron) in Fig. 5i and the tumor tissue TEM image in Fig. 5j, k confirmed that the SSM-PEG accumulated in the tumor site. The in vivo biocompatibility of SSM-PEG was also monitored via the blood routine examination, main organ pathological examination, and bio-distribution in vivo (Supplementary Figs. 28–30); all the results indicated that SSM-PEG has good biosafety in vivo. Thus, according to the MRI results on the xenograft model, it was proved that SSM-PEG could visualize the tumor site in vivo well.

### MRI detection for the metastatic model

**Fig. 6:**
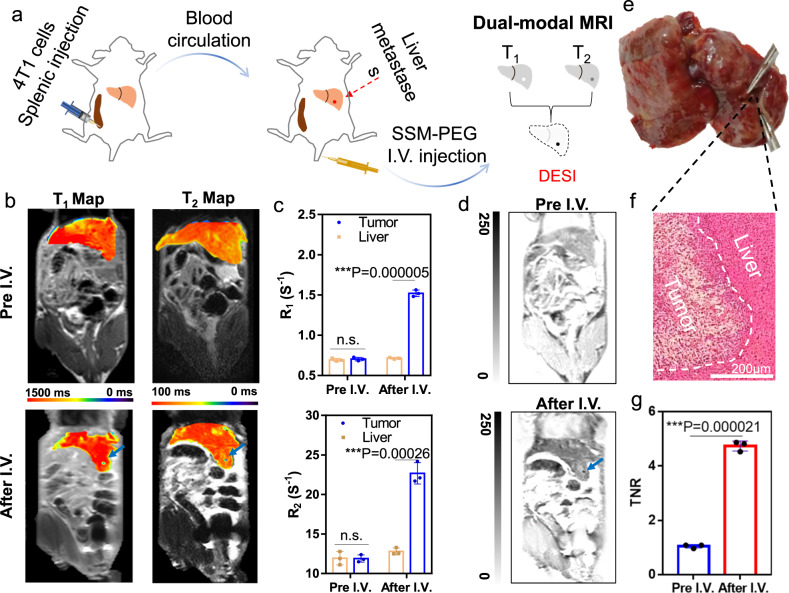
SSM-PEG (PEG-modified SPIO@SiO_2_@MnO_2_) for MRI trace of tiny liver metastases in vivo. **a** Illustration of the construction and MRI diagnosis processes of the metastatic model. **b** T_1_ and T_2_ map images and **c** *R*_1_ and *R*_2_ at the liver metastasis of the mice before I.V. injection and 2 h after the injection of SSM-PEG. T_1_ and T_2_ Map are representative data from three mice samples. R_1_ and R_2_ Data are presented as mean ± SD from three independent mice samples. **d** T_1_ and T_2_ dual-modal MRI images of the liver metastasis were processed by DESI method, which were taken without the use of contrast agent and 2 h after the injection of SSM-PEG. Representative data are shown from three mice samples. **e** Ex vivo image of the liver with a tiny metastasis. Representative data are shown from three mice samples. **f** H&E stain of the slices of the liver metastasis. Representative data are shown from three mice samples. **g** TNR quantified from the DESI processed images in d and e. Data are presented as mean ± SD from three independent mice samples. The two-tailed Student’s *t*-test is employed for the statistical analysis. *P < 0.05; **P < 0.01; ***P < 0.001. Source data are provided as a Source Data file.

Considering that SSM-PEG can bring significant MRI contrast between the tumor and normal tissue, we attempted to use it to trace the tiny liver metastases. The metastatic model was established by splenic injection of 4T1 breast cancer cells (Fig. 6a). By MRI monitoring, we found that liver micro-metastatic tumors were well-formed in most of the mice as early as 1 week post-injection (Supplementary Fig. 31). However, some of the micro-metastatic tumors had grown big in size. In one case, after the intravenous injection of SSM-PEG, a micro-metastasis (diameter less than 0.5 mm) was visible by both the T_1_-weighted and T_2_-weighted images, which could be more clearly discriminated after DESI treatment. However, without the injection of SSM-PEG, the same micro-metastasis could not be observed even using the DESI treatment (Fig. 6d). By using the SSM-PEG contrast agent, both the signals on T_1_ and T_2_ map images at the liver metastasis were significantly enhanced in comparison with the normal liver tissue (Supplementary Fig. 32 and Fig. 6b–d), while there was almost no contrast difference between the normal and tumor tissues without the injection of the SSM-PEG. The signal ratio from tumor-to-normal liver tissue (TNR) of SSM-PEG was 4.7 times via the DESI method (Fig. 6g). Ex vivo microscopies and the H&E staining image of liver tissues in Fig. 6e, f confirmed the presence of metastatic lesions of approximately 0.5 mm. This experiment proved that the high sensitivity of the SSM-PEG contrast agent made the recognition of metastatic foci in the liver possible.

### MRI detection for inflammation

**Fig. 7:**
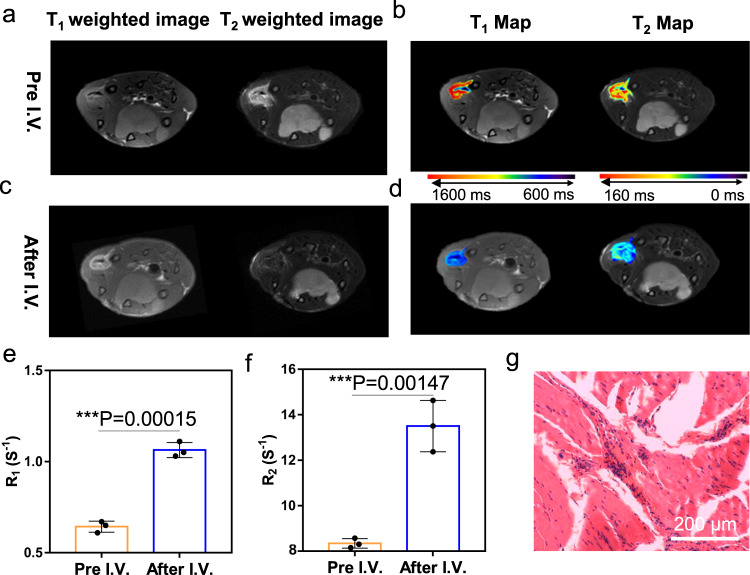
SSM-PEG (PEG-modified SPIO@SiO_2_@MnO_2_) for MRI diagnosis of inflammation in vivo. **a** T_1_ and T_2_ weighted images and **b** T_1_ & T_2_ Map of muscle inflammation without the use of contrast agents. Representative data are shown from three mice samples. **c** T_1_ and T_2_ weighted images and **d** T_1_ and T_2_ Map of muscle inflammation of the rats receiving I.V. injection of SSM-PEG. Representative data are shown from three mice samples. **e** *R*_2_ and **f** *R*_1_ at the muscle inflammation sites before and after 2 h injection of SSM-PEG contrast agents. Data are presented as mean ± SD from three independent mice samples. **g** H&E stain of tissue slices taken from the muscle inflammation sites. Representative data are shown from three mice samples. The two-tailed Student’s *t*-test is employed for the statistical analysis. *P < 0.05; **P < 0.01; ***P < 0.001. Source data are provided as a Source Data file.

In the inflammation tissue, anaerobic glycolysis, lactic acid accumulation, hypoxia, and hypochlorous acid production by activated neutrophils contribute to local acidosis, also leading to an acidic microenvironment^35,36^. So, the pH value of the microenvironment was strongly associated with inflammation severity. On the other hand, numerous reports have revealed that nanoparticles could also accumulate in the inflammation site^37,38^. Therefore, we propose that dual-activatable contrast agent SSM-PEG could also be used for inflammation diagnosis. The muscle inflammation model was established by injecting turpentine into the thigh muscle of rats. After the inflammation model was established, SSM-PEG was intravenously injected for MRI detection. As shown in Fig. 7a–d, SSM-PEG through the I. V. injection could accumulate and present higher signals on T_1_ and T_2_ weighted and map images in the inflammation tissue in comparison with that of the normal tissue. Conversely, the inflammation foci could not be clearly observed by both the T_1_-weighted and T_2_-weighted images before the injection of SSM-PEG. The *R*_1_ and *R*_2_ values at the muscle inflammation sites before and after the injection of SSM-PEG contrast agents were summarized in Fig. 7e, f. Significant MRI signal changes were revealed. The H&E stain slice confirmed the presence of inflammation in muscle tissue (Fig. 7g). All these results proved that SSM-PEG also has the potential to monitor tissue inflammation.

## Discussion

In summary, to improve the diagnostic sensitivity of the MRI technique, we developed an MRI contrast agent, SSM, with a core/shell/shell structure and showing T_1_ and T_2_ dual-activatable signals. In this structure, the MnO_2_ layer is pH-sensitive, which plays a crucial role in the signal transductions from normal tissue to diseased tissue. In the normal physiological condition, we revealed that the MnO_2_ shell would shield the magnetic field of the SPIO core and consequently reduce the T_2_ signal. Moreover, the diminishing effect of the MnO_2_ layer on the T_2_ signal of the SPIO core was highly dependent on the thickness of the intermediate SiO_2_ layer. The maximal decrease (~12 times) in T_2_ relaxation rates could be reached if the thickness of SiO_2_ was reduced to 4 nm. Based on the excellent MRI sensitivity, SSM was successfully applied to trace the tiny liver metastases of approximately 0.5 mm and to accurately monitor tissue inflammation. Overall, this research provides a design idea for a contrast agent that can provide high sensitivity for lesion detection, which shows great clinical significance in early diagnosis, prognosis, prediction, and monitoring of the recurrence of diseases.

## Methods

### Materials

Chemicals and solvents were used as received without further purification unless specified otherwise. Iron chloride, sodium oleate, oleic acid, Igepal CO-520, tetraethyl orthosilicate (TEOS), and 1-octadecene (ODE) were obtained from Aladdin Reagent (Shanghai) Co., Ltd Potassium permanganate (KMnO_4_) and Manganese sulfate (MnSO_4_) were purchased from Sinopharm Chemical Reagent Co., Ltd mPEG_5K_-NH_2_ and RhB-PEG_5K_-NH_2_ were obtained from Shanghai Ponsure Biotech, Inc. Other commonly used solvents were obtained from Aladdin Reagent (Shanghai) Co., Ltd. Prussian blue iron stain kit was obtained from Sangon Biotech (Shanghai) Co., Ltd. The Cell lines 4T1 (catalog number SCSP-5056), 293T (catalog number SCSP-502), and U87(catalog number TCHu138) were purchased from Cell Bank of Chinese Academy of Sciences. Cell culture materials, such as RPMI 1640 medium, DMEM, fetal bovine serum (FBS), and streptomycin/penicillin solution, were supplied by Gibco BRL Co., Ltd (Grand Island, NY, USA). BALB/c mice, nude mice and nude rats were purchased from Vital River Laboratory Animal Technology Co., Ltd (Beijing, China). Housing: ambient temperature 20–26 °C, relative humidity 40–70%, dark/light cycle 19:00–07:00/07:00–19:00.

### Characterizations

TEM (FEI Talos F200x) was employed to observe the morphology of SPIO, SPIO@SiO_2_, and SPIO@SiO_2_@MnO_2_. The samples (1 mg mL^−1^) were made by directly dripping the nanoparticle solution onto copper grids and placed at room temperature to dry naturally. The element mapping was performed by the EDS attachment of HR TEM. Size distribution of the above nanoparticle solutions (1 mg mL^−1^) were observed by dynamic light scattering (DLS) instruments (Malvern, Nano-ZS). UV–vis absorption spectra were recorded with a Lambda 35 spectrometer (PerkinElmer, America) between 200–800 nm at 2 nm intervals. The Electron Paramagnetic Resonance (EPR) curves were recorded on a Bruker EMXplus-10/12 EPR Spectrometer. All continuous-wave EPR data were acquired under non-saturating conditions at room temperature, with an excitation microwave frequency of 9.87 GHz, microwave power of 0.6325 mW and modulation frequency of 100 kHz. Hysteresis loops were measured at room temperature using a vibrating sample magnetometer LakeShore7404. Before the test, we prepared the sample SS and SSM with the same SPIO concentration. During the test, the same volume of each type of sample was loaded into the same liquid sample holder with a cap from Lake Shore Cryotronics and filled to capacity before the measurement.

### Synthesis of superparamagnetic iron oxide (SPIO)

SPIO was prepared through the thermal decomposition of iron-oleic acid complexes at a high temperature. In a typical synthesis, iron chloride (6.5 g, 40 mmol) was first dissolved in a mixture of 80 mL ethanol and 60 mL ultrapure water in a three-neck flask (500 mL). Afterward, sodium oleate (36.5 g, 120 mmol) was added to the iron chloride solution along with 140 mL hexane. After the complete dissolution, the reaction solution was heated to ~ 70 °C for 4-h reflux, then the upper organic layer containing the iron oleate was washed three times with ultrapure water and placed under a vacuum at 60 °C overnight. Iron oleate (3 mmol, 2.7 g) and oleic acid (1.65 mmol, 0.468 g) were added into a three-neck flask with 15 g ODE as solvent. The mixture was stirred under Argon flow at 100 °C. After 30 min, the mixture was heated to 300 °C at a rate of 18 °C/min. After 1 h, the reaction solution was quickly cooled, and the resulting products were precipitated by acetone, then purified three rounds by acetone/hexane. The final product was dispersed in cyclohexane.

### Synthesis of SPIO@SiO_2_

A reverse microemulsion method was used to prepare SPIO@SiO_2_ core−shell nanoparticles. Typically, 0.25 g Igepal CO-520 was dispersed in 5 mL of cyclohexane, and the resultant solution was sonicated for 5 min. Then, the solution of 12-nm SPIO (10 mg mL^−1^, 100 μL) was added to the above solution and sonicated for 5 min. Subsequently, 50 μL of ammonium hydroxide was added and sonicated for another 5 min. Finally, TEOS (2 μL) was added, and the mixture was placed in a flip shaker. The reaction time was set as 2, 6, and 16 h to control the shell thickness of SiO_2_. Finally, 2 mL ethanol was added to stop the reaction, and the products were centrifuged and washed three times by ethanol and ultrapure water, respectively. The obtained SPIO@SiO_2_ was finally dispersed in 1 mL ultrapure water for use.

### Synthesis of SPIO@SiO_2_@MnO_2_ (SSM)

As prepared SPIO@SiO_2_ (1 mL) was mixed with the KMnO_4_ solution (1 mg mL^−1^ in water, 1.58 mL), and the mixture was stirred for 5 min. Then, the MnSO_4_ solution (1 mg mL^−1^, 2.26 mL) was added dropwise into the above mixture. After 4 h, the products were collected by centrifugation and then washed by water three times. Finally, the product was placed on the magnetic separation rack overnight, and then the separated SPIO@SiO_2_@MnO_2_ product was dissolved in 1 mL ultrapure water for future use. For the T_1_ control group, MnO_2_ was prepared using the same method just without the addition of SPIO@SiO_2_ at the very beginning.

### Synthesis of SPIO@SiO_2_@MnO_2_ -PEG (SSM-PEG)

To prepare SPIO@SiO_2_@MnO_2_-PEG, 2 mg of mPEG-NH_2_ was added to a solution of SPIO@SiO_2_@MnO_2_ (1 mg mL^−1^, 1 mL), and then the mixture was placed in a flip shaker for 24 h. The resulting SPIO@SiO_2_@MnO_2_ -PEG product was purified by magnetic separation to remove the unreacted mPEG-NH_2_. The biocompatible T_1_ control group MnO_2_-PEG was prepared using the same method by the addition of MnO_2_ at the very beginning.

### MRI performance

The T_1_-weighted imaging (T_1_WI), T_2_-weighted imaging (T_2_WI), T_1_ map, and T_2_ map of SSM with different SiO_2_ thickness and in different pH environment (incubated 24 h) were measured on a 3.0 T MRI scanner (GE signa HDx, USA).

The acquisition parameters were set as:

T_1_WI, repetition time (TR) = 500 ms, echo time (TE) = 12 ms, slice thickness = 1 mm, slice spacing = 1 mm, FOV = 10 × 10 cm, matrix = 256 × 256;

T_1_ Map images, TR = 4.0 ms, TE = 2.0 ms, slice thickness = 1 mm, slice spacing = 1 mm, FOV = 10 × 10 cm, matrix = 256 × 256;

T_2_WI, TR = 2500 ms, TE = 60 ms, slice thickness = 1 mm, slice spacing = 1 mm, FOV = 10 × 10 cm, matrix = 256 × 256;

T_2_ Map images, TR = 1000 ms, TE = 12 − 180 ms, slice thickness = 1 mm, slice spacing = 1 mm, FOV = 10 × 10 cm, matrix = 256 × 256.

Quantitative T_1_ and T_2_ relaxation maps were reconstructed from datasets via Paravision 4 software. The same method was applied for the pH sensitivity evaluation of SSM in different pH conditions.

### In vitro MR imaging

To evaluate the pH sensitivity in vitro, We chose different cell lines to simulate the different pH environments. Renal epithelial cells (293T), breast cancer cells (4T1), and glioma cells (U87) were seeded in 6 cm cell culture dishes (1× 10^6^ cells per well) and incubated for 12 h, respectively. SSM-PEG (0.1 mg mL^−1^) was added to each well and incubated for 2 h. the cells were washed three times with PBS, and incubate with fresh medium for another 4 h. After incubation, cells were digested and centrifuged for 5 min at 175 × *g* and resuspended in 1 mL agarose (0.5 wt.%). The T_1_ and T_2_-weighted MR images were acquired using a 3.0 T MR system. For T_2_ Map images, the MRI parameters were TR = 1000, TE = 12–180 ms, FOV = 10 × 10 cm, matrix = 256 × 256. For T_1_ Map images, the MRI parameters were TR = 4.0 ms, TE = 2.0 ms, FOV = 10 × 10 cm, matrix = 256 × 256.

The mean T_1_ and T_2_ relaxation times (ms) were measured for each sample. Quantitative T_1_ and T_2_ maps were reconstructed from datasets using MATLAB software.

To investigate the time dependence properties, 293T cells and 4T1 cells were seeded in 6 cm cell culture dishes (1 × 10^6^ cells per well) and incubated for 12 h, respectively. SSM-PEG (0.1 mg mL^−1^) was added to each well and incubated for different times (0.5, 1, 2, 4, 8, and 16 h). And then, the MRI performances were measured according to the method mentioned above.

### Stability of SPIO@SiO_2_@MnO_2_-PEG (SSM-PEG)

The size distribution and zeta potential of SSM before and after PEGylation were observed by dynamic light scattering (DLS) instruments (Malvern, Nano-ZS). The stability of SSM-PEG (0.1 mg mL^−1^) was measured in 10% FBS solution by DLS.

### Hemolysis test of SPIO@SiO_2_@MnO_2_-PEG (SSM-PEG)

The hemolysis of SPIO@SiO_2_@MnO_2_-PEG (SSM-PEG) was investigated using fresh blood from healthy mice (female BALB/c mice, aged 6–8 weeks). The red blood cells (RBCs) were collected by centrifugation at 175 × *g* for 10 min, washed three times with PBS, and then brought to a final concentration of 2% (v/v) in PBS. Two hundred milliliters of erythrocyte suspension was mixed with different concentrations (0.05 and 0.10 mg mL^−1^) of SSM and SSM-PEG, respectively, and incubated for 4 h at 37 °C in an incubator shaker. The mixtures were centrifuged at 175 × *g* for 5 min, and the supernatants (100 μL) of all samples were transferred to a 96-well plate. Free hemoglobin in the supernatant was measured by the absorbance at 540 nm using a microplate reader (Cytation 3 microplate reader, BioTek Instruments, Inc., Winooski, USA). RBC incubated with DI water and PBS buffer were used as the positive and negative controls, respectively.

### Cytotoxicity

To evaluate the cytotoxicity of SSM before and after PEGylation, 4T1 cells seeded in 96-well plates were pre-incubated with different concentrations of SSM and SSM-PEG (normalized by the concentration of iron: 0.2, 0.4, 2.0, 4.0, 20, 40 μM). After a 24-h incubation, the cells were gently washed by PBS buffer three times to remove the non-internalized nanoparticles. The cell viabilities were determined by MTT assay, and the results were presented as average ± SD (*n* = 3).

### Pharmacokinetic analyses

The pharmacokinetics of SSM and SSM-PEG were systematically investigated using Inductively Coupled Plasma Mass Spectrometry (ICP-MS, Agilent 7700x) for the quantified amount of Fe in the blood samples. Briefly, the blood samples were collected at different time points (5, 10, 20 30 min, 1, 2, 4, and 24 h) after the administration of SSM and SSM-PEG (10 mg kg^−1^) via intravenous injection (female BALB/c mice, aged 6–8 weeks, 3 mice per group).

### In vivo MR imaging of subcutaneous xenograft model

All animal experiments were in accord with Institutional Animal Use and Care Regulations, approved by the Laboratory Animal Ethics Committee of the Southern University of Science and Technology (No. SUSTC-JY2019068) and Shanghai General Hospital. To establish subcutaneous tumors in mice, 4T1 cells were harvested by centrifugation, washed, and resuspended in PBS (2 × 10^7^ cells mL^−1^). A 100-μL cell suspension containing 2 × 10^6^ cells was then injected subcutaneously into the right flank of the female BALB/c mice (aged 6–8 weeks, 1 site per mice). When tumors were well established and had reached diameters of 6–8 mm (tumors were measured with vernier caliper, and the volume was calculated by *V* = 0.5 × length × width^2^; for both single and bilateral flank tumor models, mice were sacrificed when the total tumor burden exceeded 2000 mm^3^), the mice were used for in vivo MR imaging. Mice bearing with 4T1 breast tumor were scanned on a 3.0 T MR system with a high-resolution animal coil at 0, 1, 6, and 12 h after tail vein injection of the SPIO-PEG, MnO_2_-PEG, and SSM-PEG (100 μL each, normalized by SPIO concentration of 1 mg mL^−1^, SPIO-PEG was set as the T_2_ control group, and MnO_2_-PEG was set as the T_1_ control group, *n* = 3). All mice were imaged under the T2WI spin-echo sequence (TR/TE 2000/60 ms) and T_1_WI spin-echo sequence (TR/TE 450/12 ms) with a 256 × 256 matrix size. The mean T_2_-weighted signal intensities and T_1_-weighted signal intensities were measured for each tumor. For T_2_ Map images (TR = 1000, TE = 12–180 ms, FOV = 12 × 12 cm, matrix = 256 × 256) and T_1_ Map images (TR = 4.0 ms, TE = 2.0 ms, FOV = 12 × 12 cm, matrix = 256 × 256) were obtained with a 3.0 T MRI. The mean T_2_ relaxation time and T_1_ relaxation time were measured for each tumor. Quantitative T_2_ maps and T_1_ maps were reconstructed from datasets using MATLAB software.

### In vivo MR imaging of liver metastatic models

To prepare the liver metastatic models, the spleen was exposed through the incision, 4T1 cells (1 × 10^5^) were suspended in 50 μL PBS and injected into the edge of the spleen (female nude mice at the age of 6–8 weeks), then the mouse was sutured. Two weeks later, these mice were collected for in vivo MR imaging. Mice bearing with 4T1 breast tumor were scanned on a 3.0 T MR system with a high-resolution animal coil at 0 h and 2 h after tail vein injection of the SSM-PEG (100 μL, by SPIO concentration of 1 mg mL^−1^, *n* = 3). All mice were imaged under the T_2_WI spin-echo sequence (TR/TE 2000/60 ms) and T_1_WI spin-echo sequence (TR/TE 450/12 ms) with a 256 × 256 matrix size. The mean T_2_-weighted signal intensities and T_1_-weighted signal intensities were measured for each tumor (Smean). For T_2_ Map images (TR = 1000, TE = 12–180 ms, FOV = 12 × 12 cm, matrix = 256 × 256) and T_1_ Map images (TR = 4.0 ms, TE = 2.0 ms, FOV = 12 × 12 cm, matrix = 256 × 256) were obtained with a 3.0 T MRI. The mean T_2_ relaxation time and T_1_ relaxation time were measured for each tumor. Quantitative T_2_ Maps and T_1_ Maps were reconstructed from datasets using MATLAB software.

### In vivo MR imaging of inflammation model

Female nude rats at 4 weeks of age were obtained from Charles River Laboratory Animal Technology Co., Ltd. (Beijing, China). To establish inflammation model in rats, turpentine (20 μL) was injected into the thigh muscle of rats. After 1 week, SSM-PEG was intravenously injected, and the MRI measurements were performed (*n* = 3). All rats were imaged under the T_2_WI spin-echo sequence (TR/TE 2000/60 ms) and T_1_WI spin-echo sequence (TR/TE 450/12 ms) with a 256 × 256 matrix size. The mean T_2_-weighted signal intensities and T_1_-weighted signal intensities were measured for each tumor (Smean). For T_2_ Map images (TR = 1000, TE = 12–180 ms, FOV = 12 × 12 cm, matrix = 256 × 256) and T_1_ Map images (TR = 4.0 ms, TE = 2.0 ms, FOV = 12 × 12 cm, matrix = 256 × 256) were obtained with a 3.0T MRI. The mean T_2_ relaxation time and T_1_ relaxation time were measured for each tumor. Quantitative T_2_ Maps and T_1_ Maps were reconstructed from datasets using MATLAB software.

### H&E staining and Prussian blue staining

After humanely killing the mice, we collected the major organs and tumors, which were fixed in 4% paraformaldehyde. The organs were then sliced and stained by H&E (Hematoxylin-Eosin staining) to evaluate the systemic toxicity of the contrast agents. The tumor was also sliced and stained by Prussian Blue, which was performed to detect iron uptake in cells.

### Blood routine tests

The blood of healthy mice and the tumor-bear mice with the injection of SSM-PEG were collected and proceeded to blood routine tests on an automatic hematology analyzer (DF52Vet, Dymind Biotech., China).

### Statistics and reproducibility

All of the experimental data were obtained in triplicate unless otherwise mentioned and are presented as mean ± SD. Statistical comparison by analysis of variance was performed at a significance level of *P* < 0.05 based on a Student’s *t*-test.

### Reporting summary

Further information on research design is available in the Nature Portfolio Reporting Summary linked to this article.

## Supplementary information

Supplementary Information

Reporting Summary

## Data Availability

Source data are provided with this paper. Data that support the findings of this study are available within the Article, Supplementary Information or Source Data file. Source data are provided with this paper.

## Source data

Source Data
