# Supplementary material for: A pH-responsive T1-T2 dual-modal MRI contrast agent for cancer imaging
Source: Nat Commun. 2022 Dec 26;13:7948. doi: 10.1038/s41467-022-35655-x (PMC9792454; doi:10.1038/s41467-022-35655-x)
Supplement: Supplementary file 1 — Supplementary Information [file 41467_2022_35655_MOESM1_ESM.pdf]

A pH-responsive T_1-T_2 dual-modal MRI contrast agent for cancer imaging

Hongwei Lu[1†], An Chen[2†], Xindan Zhang[1], Zixiang Wei[1], Rong Cao[2], Yi Zhu[2], Jingxiong Lu[1], Zhongling Wang[2*] and Leilei Tian[1*]

[1]Department of Materials Science and Engineering, Southern University of Science and Technology, Shenzhen, Guangdong 518055, China.

[2]Department of Radiology, Shanghai General Hospital, School of Medicine, Shanghai Jiaotong University, Shanghai 200080, China

†These authors contributed equally

*Corresponding author: tianll@sustech.edu.cn; zlwang138136@126.com

Supplementary Figures and Tables

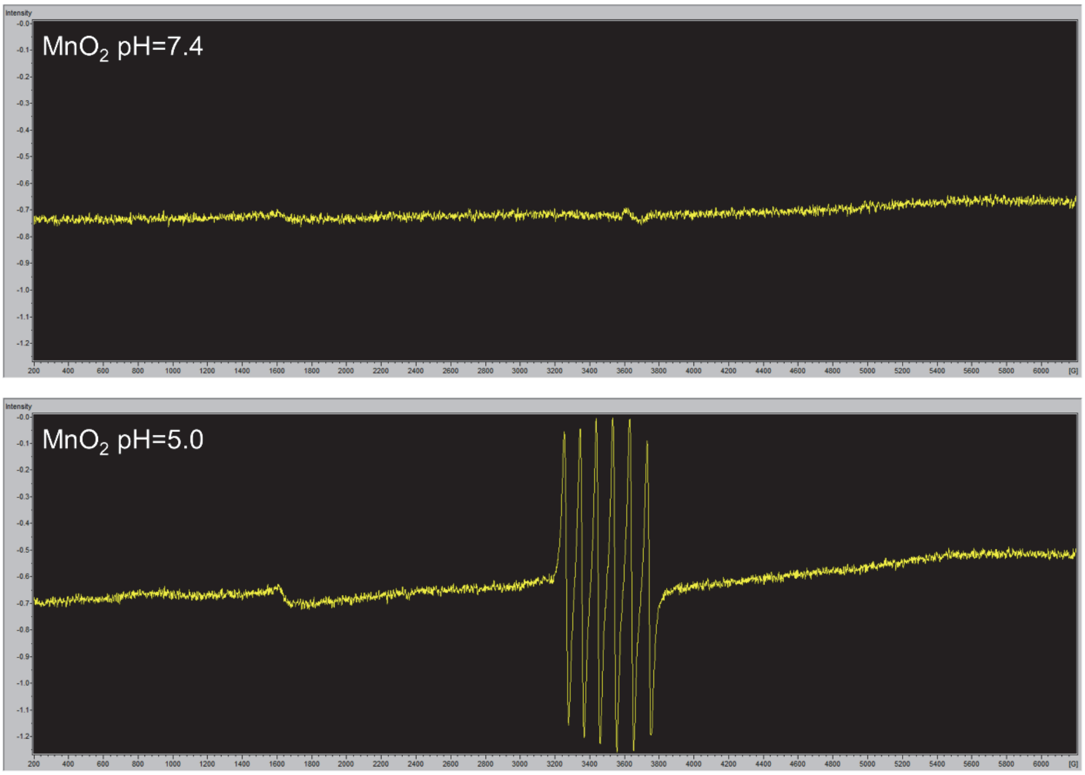

Supplementary Fig. 1 EPR spectra of MnO_2 in buffers of pH 7.4 and 5.0.

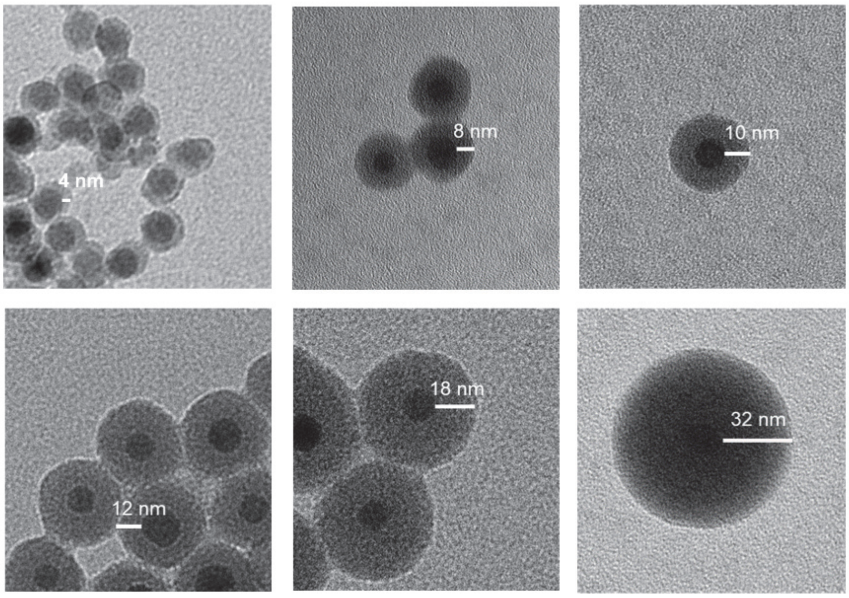

Supplementary Fig. 2 SPIO@SiO_2 with different shell thickness. Representative data are shown from three independent samples. In this method, the shell thicknesses form 4 nm to 12 nm were controlled by changing the reaction time: 2 h reaction for 4 nm SiO_2, 6 h reaction for 8 nm SiO_2, 10 h reaction for 10 nm SiO_2, and 16 h for 12 nm SiO_2. For reaching a thickness of 18 nm, the ammonium hydroxide amount was increased to 100 μL, and TEOS was increased to 4 μL, and the reaction time was elongated to 24 h. For reaching a thickness of 32 nm, 100 μL of ammonium hydroxide was used, and TEOS amount was increased to 12 μL, and the reaction time was elongated to 72 h. Source data are provided as a Source Data file.

Supplementary Table 1. The mass ratio of Fe/Mn in SSM detected by ICP-MS

SSM	Fe [ppb]	Mn [ppb]	Fe: Mn
**Thickness = 4 nm**	27.8	519.9	1:18.7
**Thickness = 8 nm**	31.6	616.2	1:19.5
**Thickness = 12 nm**	30.3	612.1	1:20.2

The MnO_2 was loaded on the surface of SPIO@SiO_2 via the disproportionated reaction between $KMnO_4$ and $MnSO_4$, we controlled the mass ratio of Fe/Mn in SSM to the same by adjusting the dosage of $KMnO_4$ (10~200 mg) and $MnSO_4$ (15~300 mg). The SSM samples with different SiO_2 thicknesses but keeping the same mass ratio of Fe/Mn (nearly 1:20) were used for all the experiments. Source data are provided as a Source Data file.

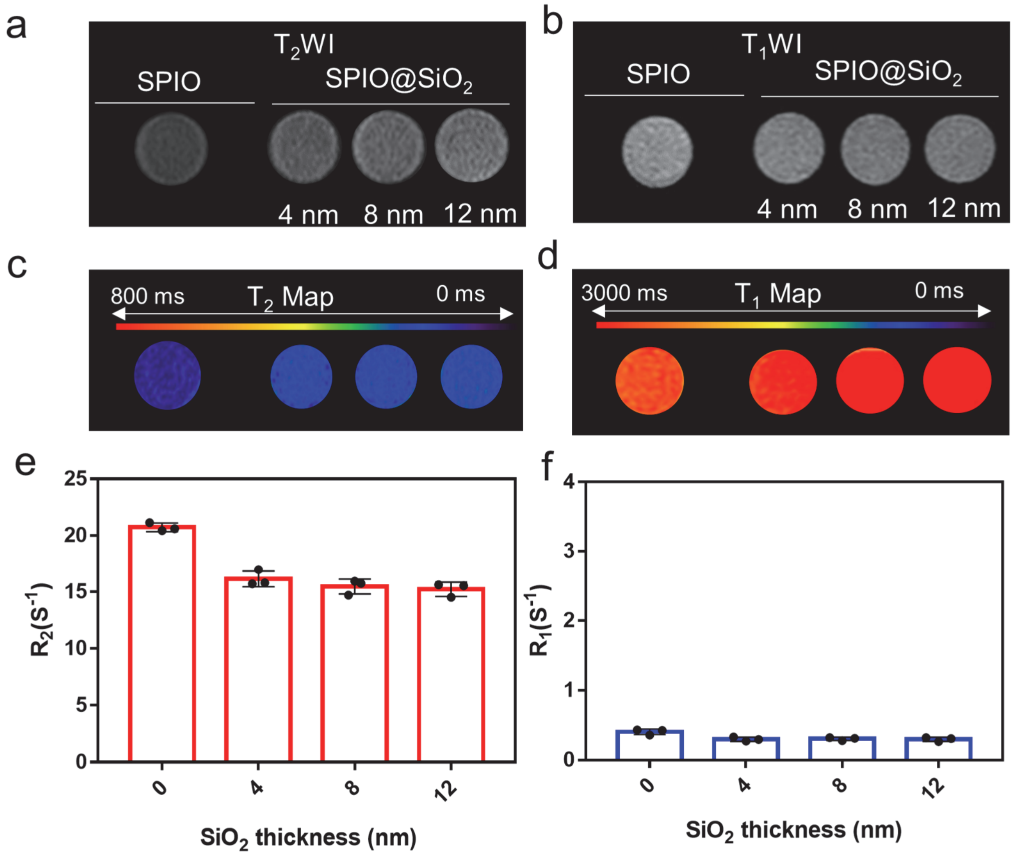

Supplementary Fig. 3 MRI performance of SPIO and SPIO@SiO_2 with different SiO_2 thicknesses (4 nm, 8 nm, and 12 nm). **a.** T_2WI; **b.** T_1WI; **c.** T_2 map; **d.** T_1 map; **e.** R_2 value; and **f.** R_1 value. SPIO@SiO_2 showed a good T_2 signal but no obvious T_1 signal; with SiO_2 thickness varied, there were no significant changes in MRI T_2 or T_1 signals. Data presented as mean ± SD from three independent samples. Source data are provided as a Source Data file.

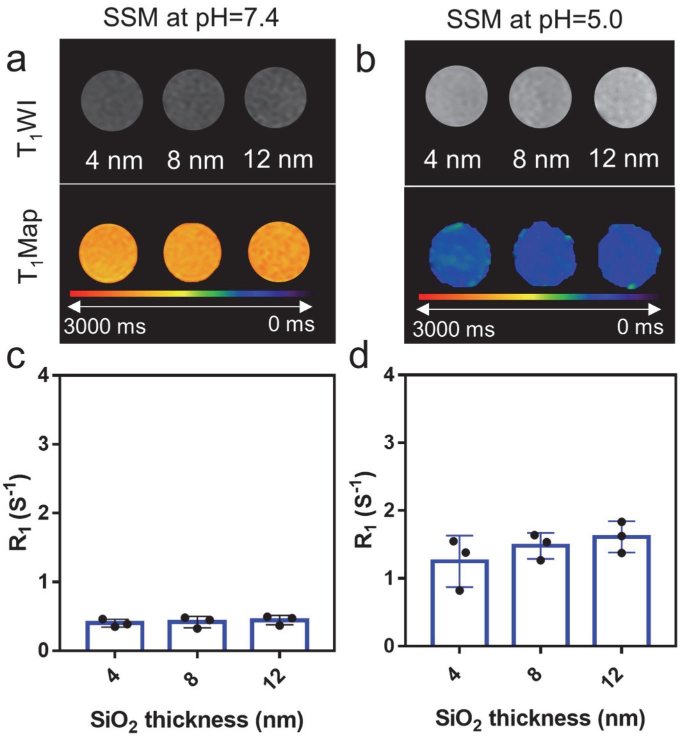

Supplementary Fig. 4 T_1 signal of SSM with different T_1-T_2 distance (shell thickness of 4 nm, 8 nm and 12 nm) at different pH environment. **a.** T_1WI & T_1 Map and **c.** R_1 value of SSM at pH 7.4; **b.** T_1WI & T_1 Map and **d.** R_1 value of SSM at pH 5.0. Data presented as mean ± SD from three independent samples. Source data are provided as a Source Data file.

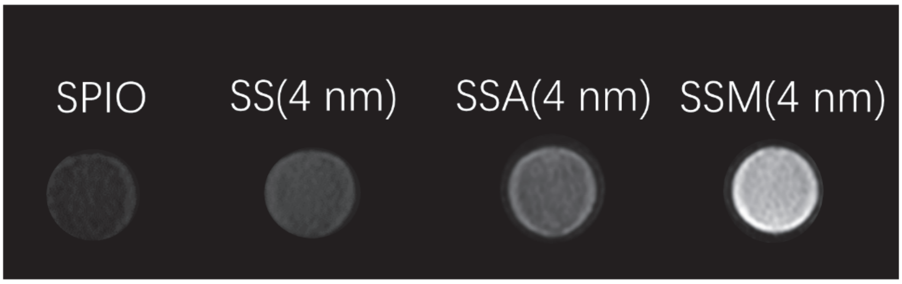

Supplementary Fig. 5 T_2WI of SPIO, SPIO@SiO_2 (SS), SPIO@SiO_2@Ag_2O (SSA) and SPIO@SiO_2@MnO_2 (SSM). Source data are provided as a Source Data file.

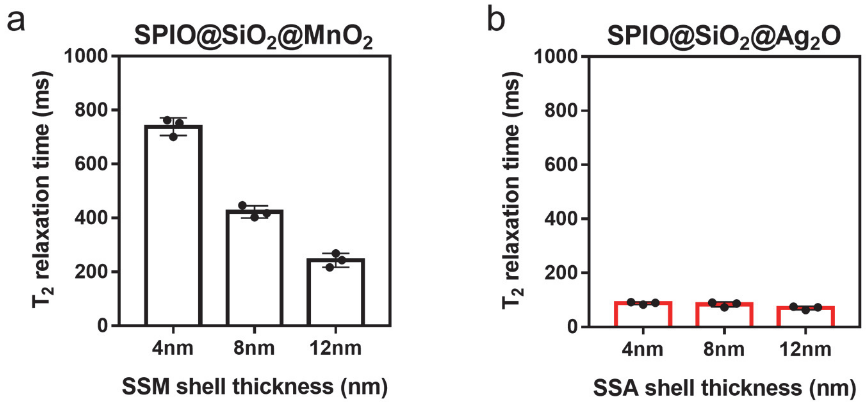

Supplementary Fig. 6 T_2 relaxation time of SPIO@SiO_2@MnO_2 (**a**) and SPIO@SiO_2@Ag_2O (**b**) with different thicknesses of the silica shell. Data presented as mean ± SD from three independent samples. Source data are provided as a Source Data file.

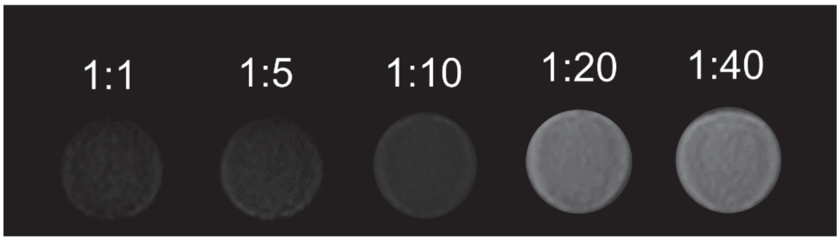

Supplementary Figure 7. T_2WI of SSM with different Fe/Mn ratio. Representative data are shown from three independent samples. Source data are provided as a Source Data file.

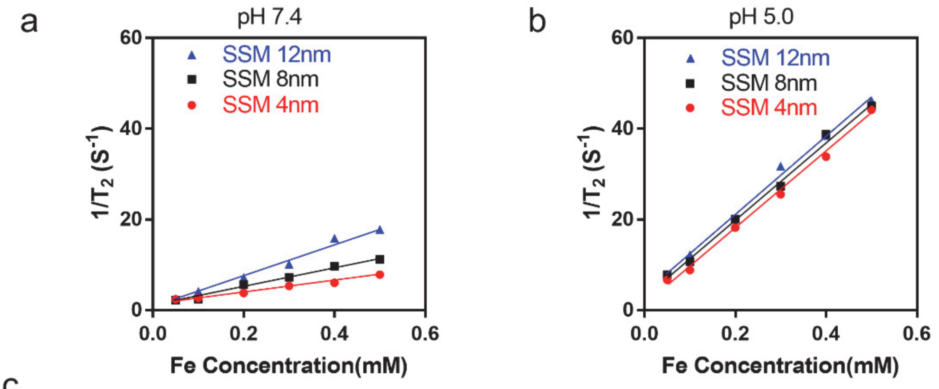

c

	r_2 at pH7.4 ($mM^{-1}S^{-1}$)	r_2 at pH5.0 ($mM^{-1}S^{-1}$)
SSM 4nm	12.08	83.48
SSM 8nm	21.06	85.57
SSM 12nm	35.38	87.28

Supplementary Fig. 8 The fitting plots of $1/T_2$ versus Fe concentration of SSM with different SiO_2 thicknesses at pH 7.4 (**a**) and 5.0 (**b**). (**c**) The table summary of the transverse relaxivity (r_2, mM^{-1} s^{-1}) of SSM with various SiO_2 thicknesses at pH 7.4 and 5.0. Representative data are shown from three independent samples. Source data are provided as a Source Data file.

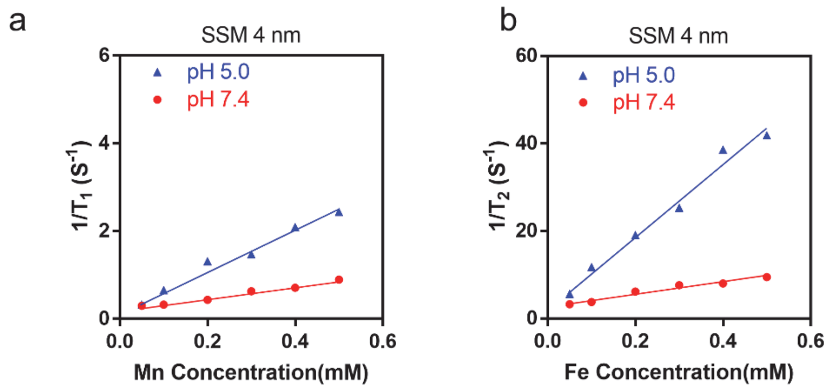

c

	$r_1 (mM^{-1}S^{-1})$	$r_2 (mM^{-1}S^{-1})$	r_2/r_1
pH 5.0	4.60	82.21	17.87
pH 7.4	1.34	13.88	10.35

Supplementary Fig. 9 The fitting plots of (**a**) $1/T_1$ versus Mn concentration and (**b**) $1/T_2$ versus Fe concentration of SSM (4 nm) at pH 7.4 and 5.0. (**c**) The table summary of the longitudinal relaxivity (r_1, mM^{-1} s^{-1}), transverse relaxivity (r_2, mM^{-1} s^{-1}), and (r_2/r_1 ratio) of SSM (4 nm) at pH value of 7.4 and 5.0. Representative data are shown from three independent samples. Source data are provided as a Source Data file.

The concentrations of Fe and Mn in SSM with the SiO_2 thickness of 4 nm were calibrated by the ICP-MS results in Supplementary Table 1. The solutions with various Fe and Mn concentrations were applied to test the T_1 longitudinal relaxation time and T_2 transverse relaxation time. The relaxivity r_1 and r_2 ($mM^{-1}s^{-1}$) were calculated from the fitting plots of $1/T_1$ or $1/T_2$ versus Fe/Mn concentration.

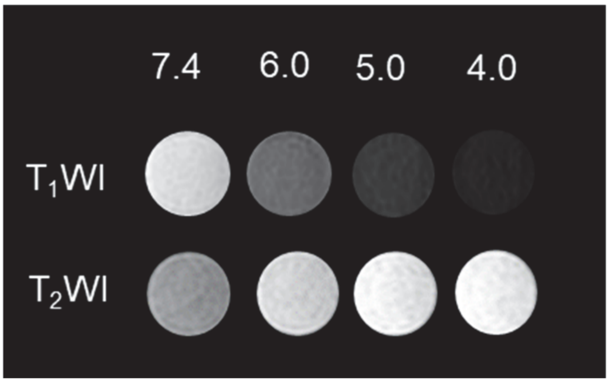

Supplementary Fig. 10 Upper lane: T_1WI of SSM in different pH environments (pH = 7.4, 6.0, 5.0, and 4.0). Lower lane: T_2WI of SSM in different pH environments (pH=7.4, 6.0, 5.0, and 4.0). Representative data are shown from three independent samples. Source data are provided as a Source Data file.

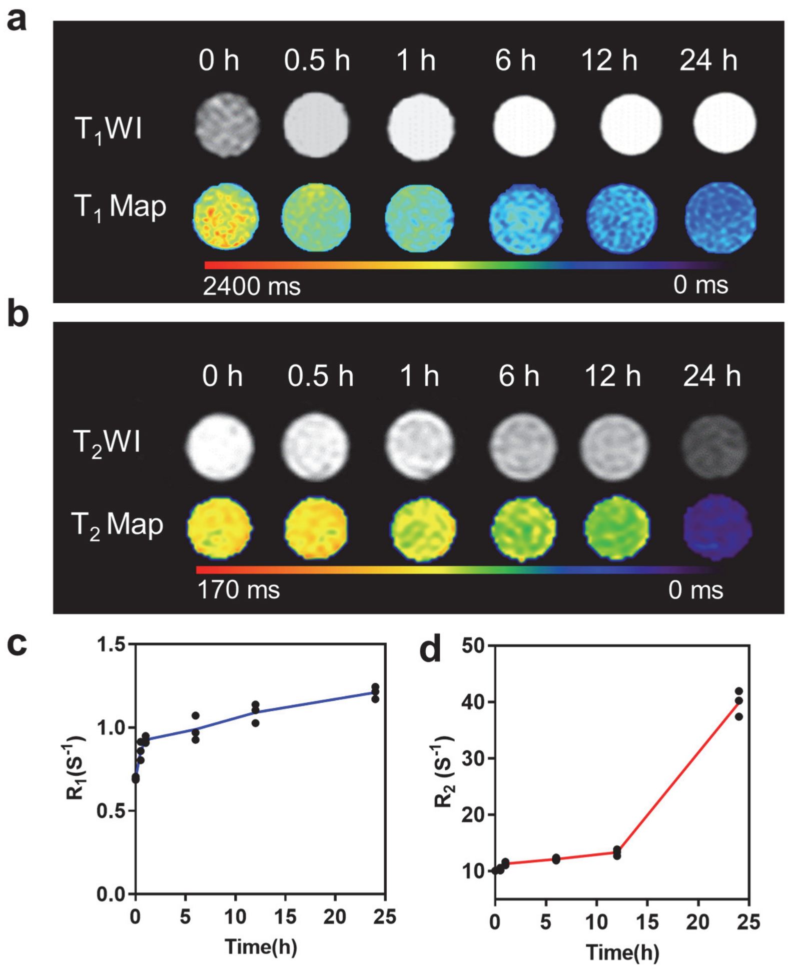

Supplementary Fig. 11 Time-dependence MRI performance of SSM at pH value of 5.0. **a.** T_1WI & T_1 Map; **b.** T_2WI and T_2 Map; **c.** R_1 value of SSM at pH value of 5.0; **d.** R_2 value of SSM at pH value of 5.0. All the results indicated that SSM performed a time-dependence MRI activity. Data presented as mean ± SD from three independent samples. Source data are provided as a Source Data file.

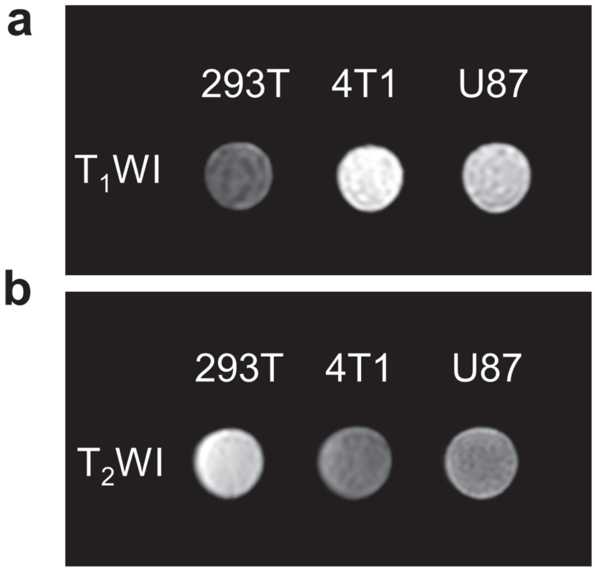

Supplementary Fig. 12 a. T_1WI of SSM after the incubations with different cell lines (293T, 4T1, and U87); **b.** T_2WI of SSM after the incubations with different cell lines (normal tissue cell 293T and cancer cell 4T1 and U87). Representative data are shown from three independent samples. Source data are provided as a Source Data file.

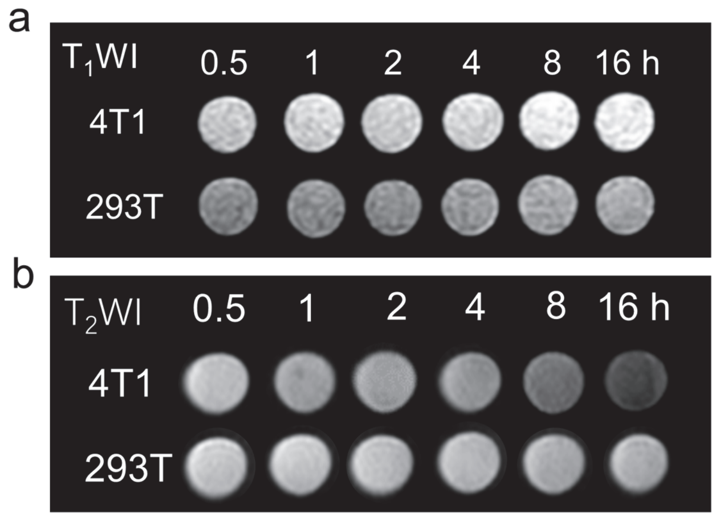

Supplementary Fig. 13 Time-dependence MRI performance of SSM with different cell lines (293T and 4T1) for 0.5, 1, 2, 4, 8, and 16 h. **a.** T_1WI images; **b.** T_2WI images. Representative data are shown from three independent samples. Source data are provided as a Source Data file.

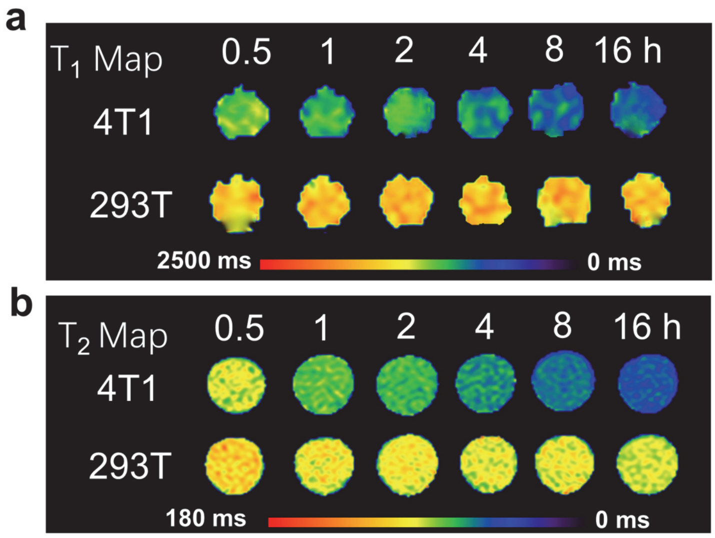

Supplementary Fig. 14 a. T_1 Maps of SSM with different cell lines (293T and 4T1) for 0.5, 1, 2, 4, 8, and 16 h; **b.** T_2 Maps of SSM with different cell lines (293T and 4T1) for 0.5, 1, 2, 4, 8, and 16 h. Representative data are shown from three independent samples. Source data are provided as a Source Data file.

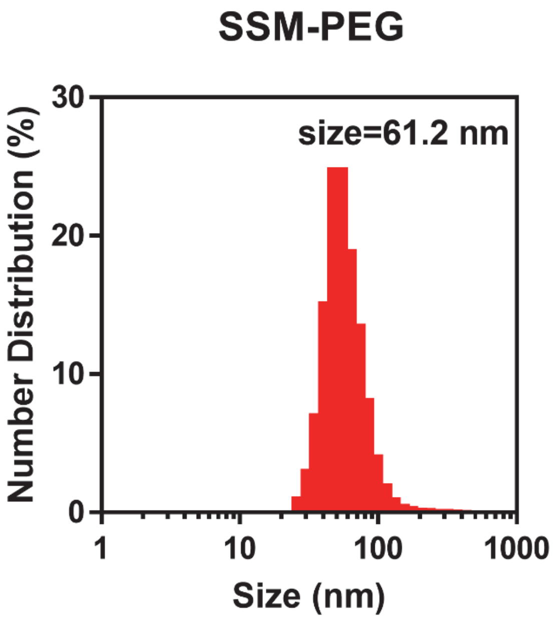

Supplementary Fig. 15 Size distribution of SSM-PEG measured by DLS. Representative data are shown from three independent samples. Source data are provided as a Source Data file.

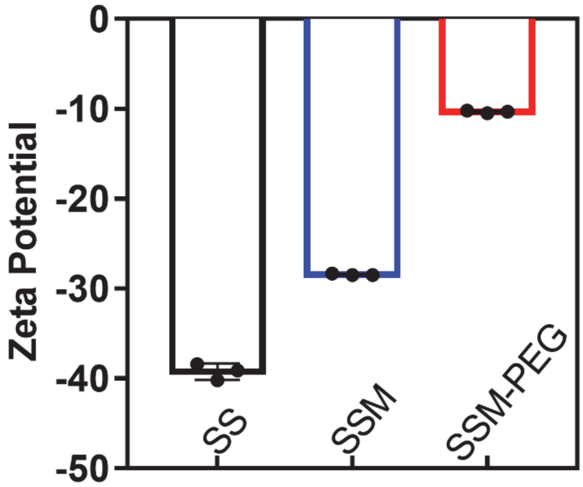

Supplementary Fig. 16 Zeta potentials of SS, SSM, and SSM-PEG. The absolute charge potential of SSM-PEG decreased obviously, as the surface charge of SSM particles could be shielded after PEGylation. This result proved the successful PEG coating. Data presented as mean ± SD from three independent samples. Source data are provided as a Source Data file.

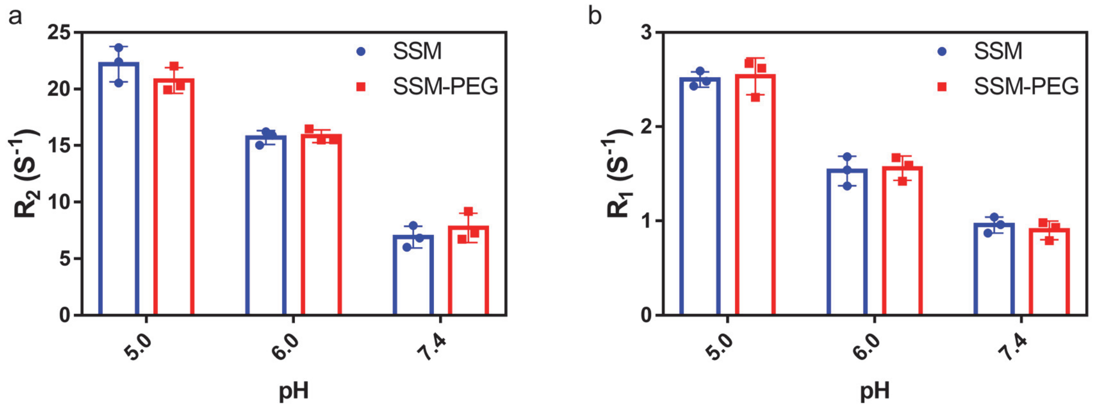

Supplementary Fig. 17 R_2 (**a**) and R_1 (**b**) values of SSM and SSM-PEG in different pH buffers. Data presented as mean ± SD from three independent samples. Source data are provided as a Source Data file.

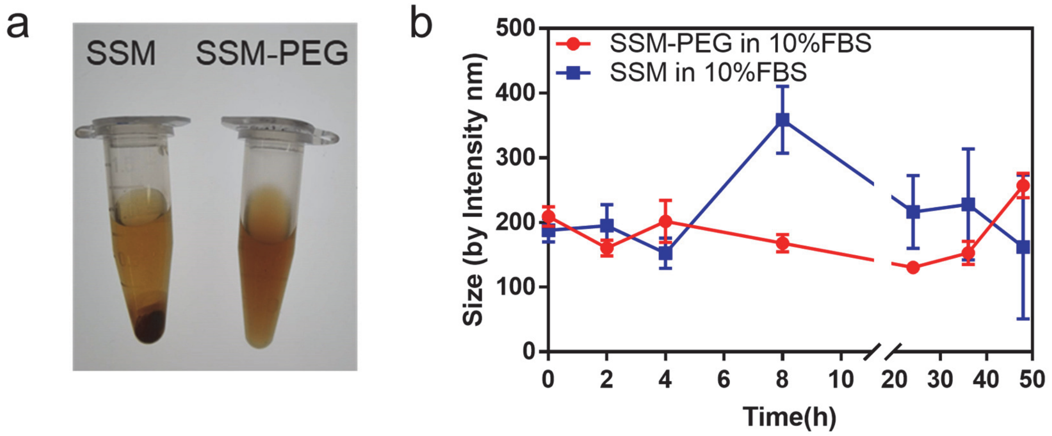

Supplementary Fig. 18 Stability of SSM-PEG in 10% FBS. **a.** A digital image of SSM and SSM-PEG incubated in 10% FBS solution after 48 h. **b.** Size monitoring data of SSM and SSM-PEG by DLS. The stability test indicated that the PEG coating on the surface of SSM enhanced its stability. The SSM-PEG could be kept stable for nearly 48 h in a 10% FBS solution. Data presented as mean ± SD from three independent samples. Source data are provided as a Source Data file.

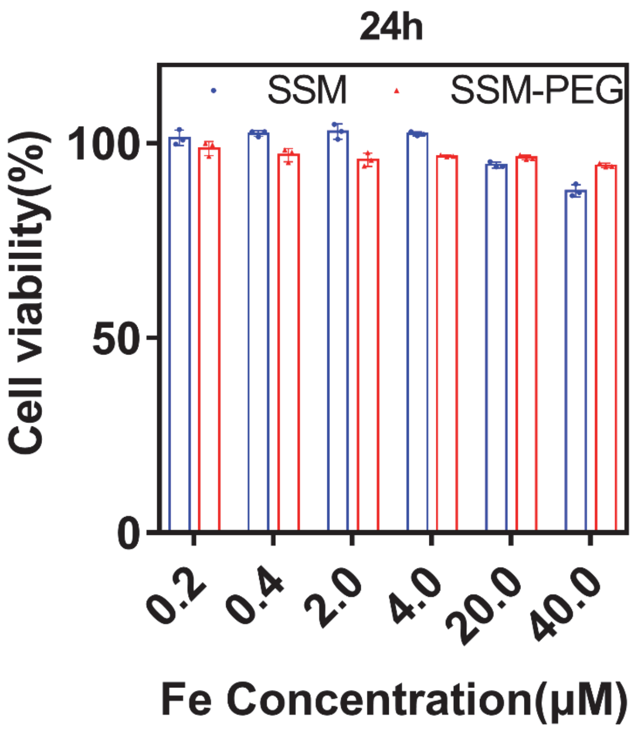

Supplementary Fig. 19 4T1 cell viabilities after the treatment with different concentrations of SSM and SSM-PEG. Data presented as mean ± SD from three independent samples. Source data are provided as a Source Data file.

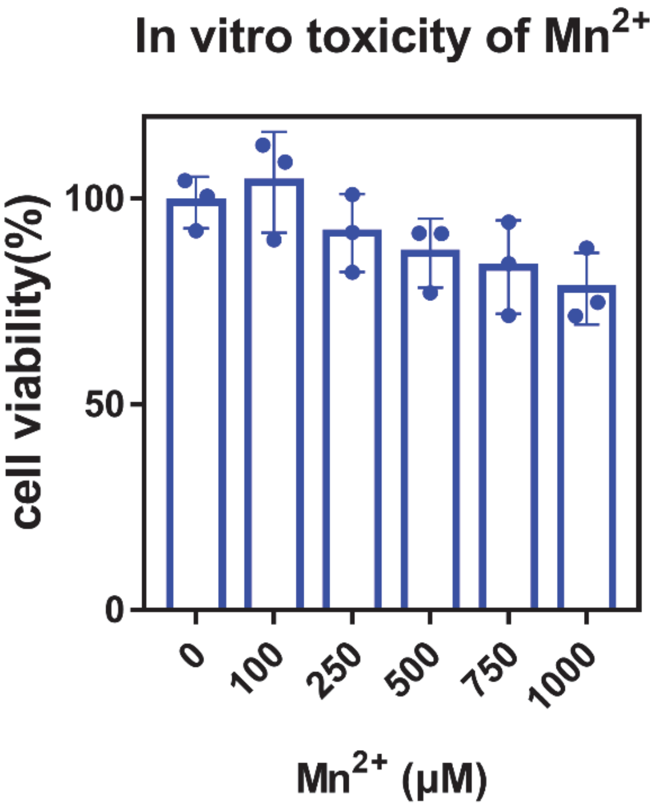

Supplementary Fig. 20 In vitro toxicity of Mn^{2+} of different concentrations. Data presented as mean ± SD from three independent samples. Source data are provided as a Source Data file.

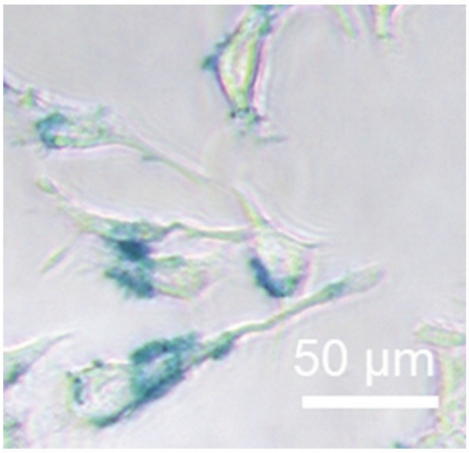

Supplementary Fig. 21 Cell uptake performance of SSM-PEG via Prussian blue staining. Source data are provided as a Source Data file. One representative data was shown from two independently repeated experiments.

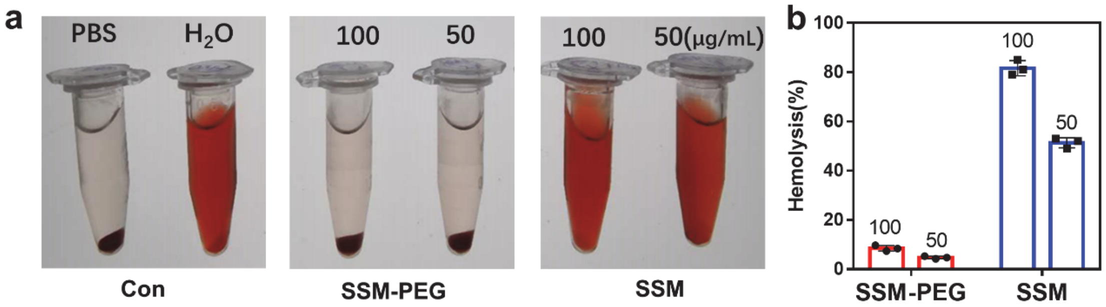

Supplementary Fig. 22 a. Digital photo of red blood cell (RBC) incubated with control, SSM-PEG and SSM. **b**. In vitro red blood cell (RBC) hemolysis of SSM and SSM-PEG at different concentrations (100 and 50 μg mL^{-1}). This result indicated that PEG coating significantly enhanced the biocompatibility of SSM, and the SSM-PEG could be used for further in vivo study. Data presented as mean ± SD from three independent samples. Source data are provided as a Source Data file.

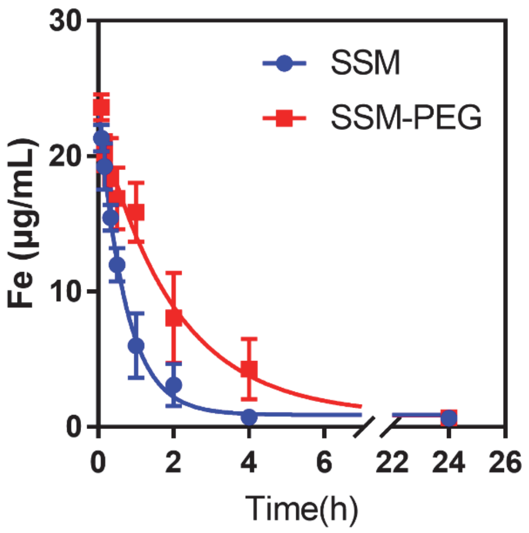

Supplementary Fig. 23 Pharmacokinetics profile of SSM and SSM-PEG. Data presented as mean ± SD from three independent samples. Source data are provided as a Source Data file.

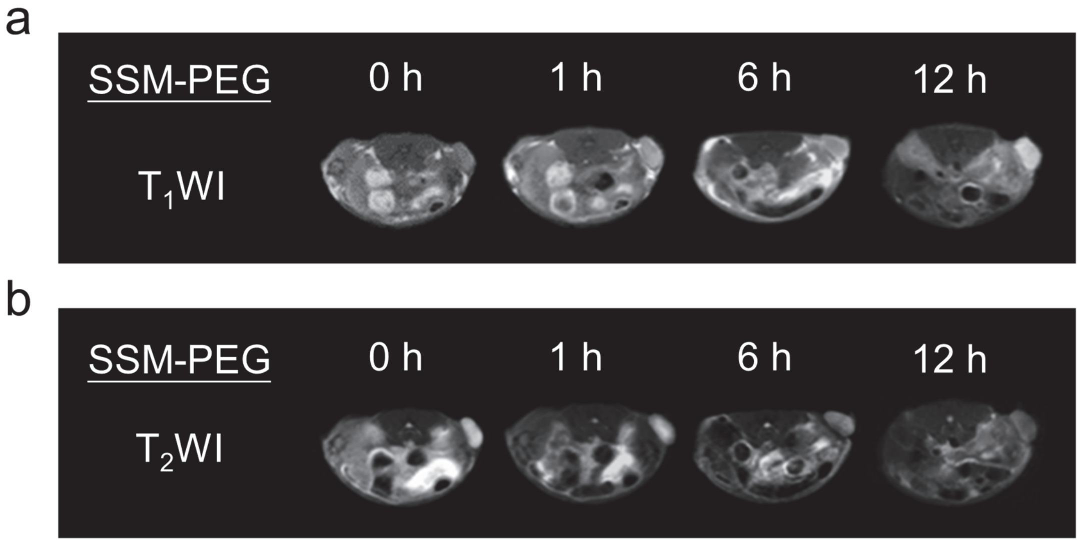

Supplementary Fig. 24 T_1 weighted image (**a**) and T_2 weighted image (**b**) of mice bearing 4T1 breast cancer xenograft following i.v. injection of SSM-PEG. Representative data are shown from three mice samples. Source data are provided as a Source Data file.

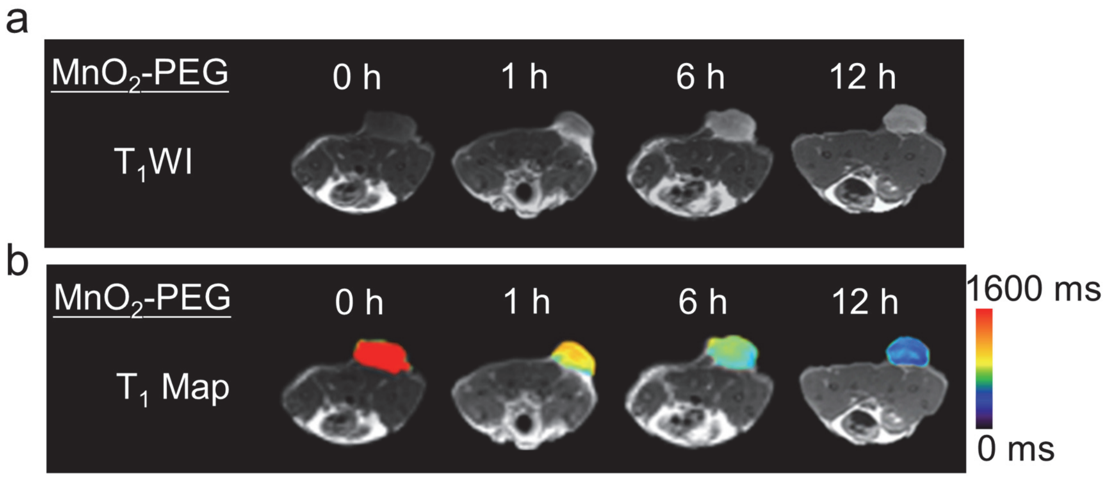

Supplementary Fig. 25 T_1 weighted image (**a**) and T_1 Map (**b**) of mice bearing 4T1 breast cancer xenograft following i.v. injection of MnO_2-PEG (T_1 control group). Representative data are shown from three mice samples. Source data are provided as a Source Data file.

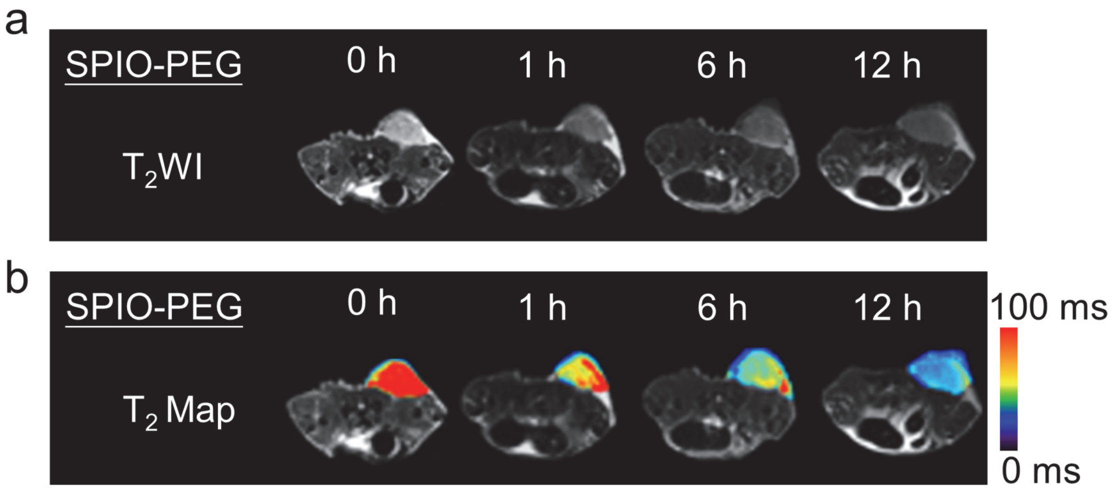

Supplementary Fig. 26 T_2 weighted image (**a**) and T_2 Map (**b**) of mice bearing 4T1 breast cancer xenograft following i.v. injection of SPIO-PEG (T_2 control group). Representative data are shown from three mice samples. Source data are provided as a Source Data file.

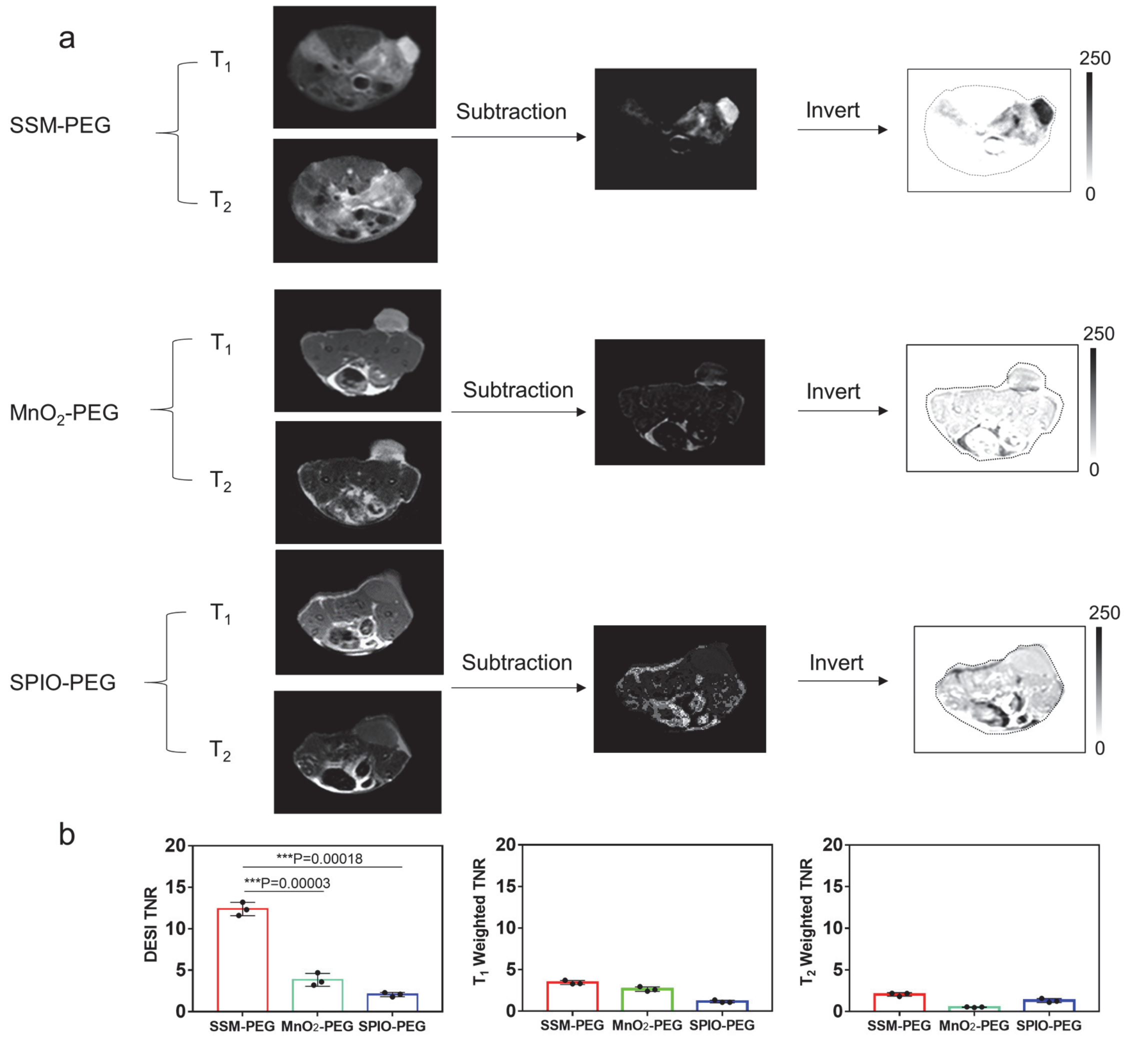

Supplementary Fig. 27 a. The detail of "Dual-contrast enhanced subtraction imaging (DESI)" imaging processing steps of SSM-PEG, the T_1 control MnO_2-PEG, and the T_2 control SPIO-PEG mentioned in Figure 4d - 4g. **b.** The tumor to normal tissue signal ratios (TNRs) quantified from DESI processed images,T_1 weighted images and T_2 weighted images. Data presented as mean ± SD from three independent samples. Source data are provided as a Source Data file.

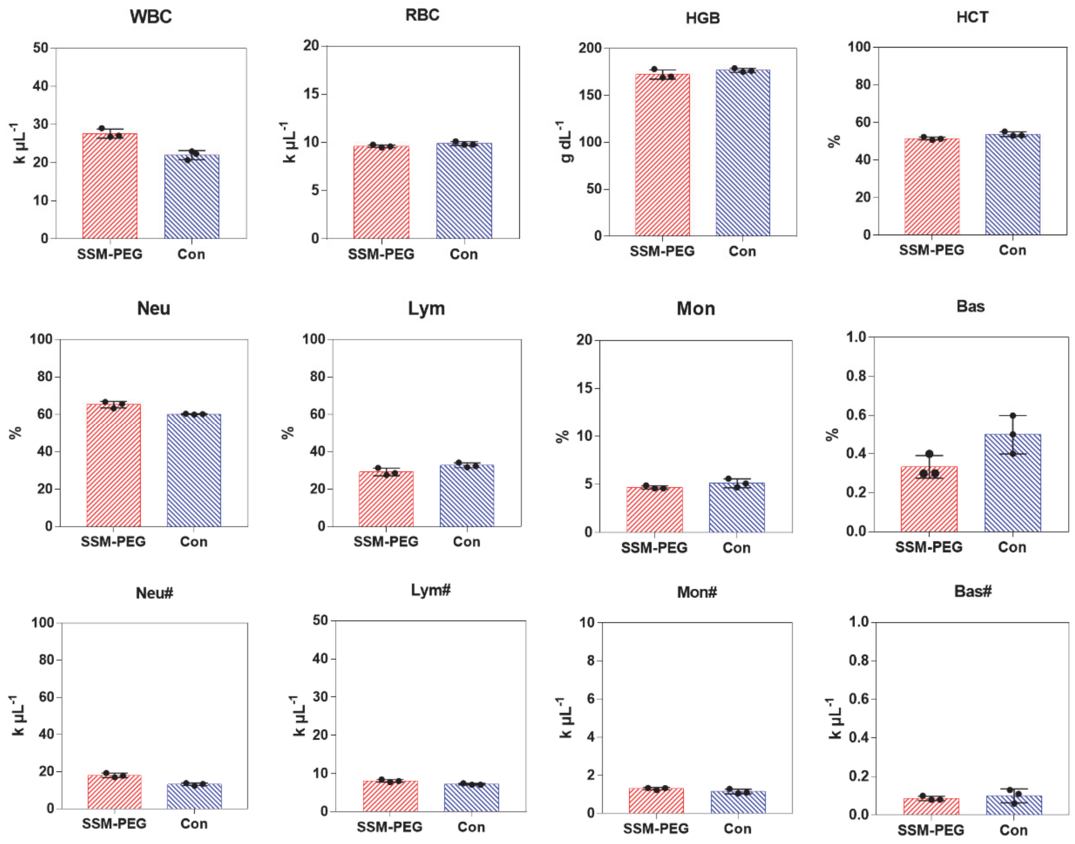

Supplementary Fig. 28 Blood routine examination of mice taken at 24 h after i.v. injection of SSM-PEG. Data presented as mean ± SD from three independent samples. Source data are provided as a Source Data file.

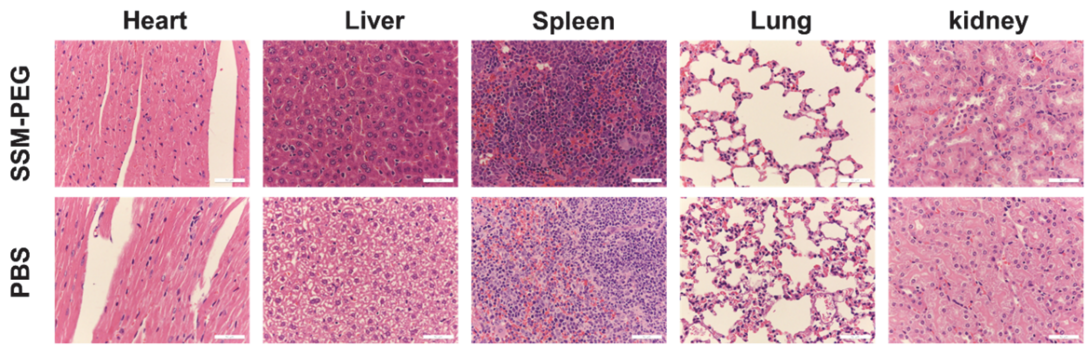

Supplementary Fig. 29 H&E staining of tissue slices of the main organs taken from mice 24 h after i.v. injection of SSM-PEG (scale bar: 50 μm). Representative data are shown from three mice samples. Source data are provided as a Source Data file.

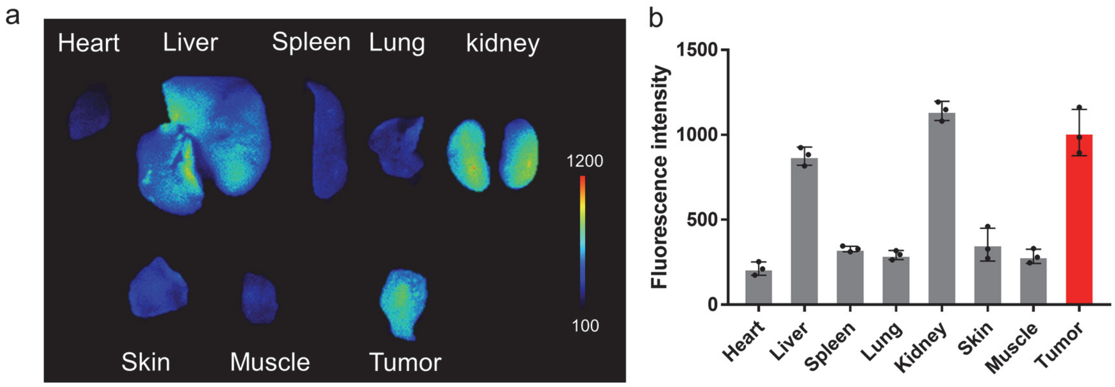

Supplementary Fig. 30 The in vivo metabolic distribution. **a.** Representative ex vivo fluorescence imaging of 4T1-bearing mice 24 h after the administration of SSM-PEG-RB (i.v. injection, dose: 5 mg kg^{-1}). **b**. Quantitative analysis of fluorescence intensity for the *ex vivo* images of different organs from the mice administrated with SSM-PEG-RB. In this experiment, we used Rhodamine B label NH_2-PEG to synthesize SSM-PEG-RB (the synthesis process was same as that of SSM-PEG). Data presented as mean ± SD from three independent samples. Source data are provided as a Source Data file.

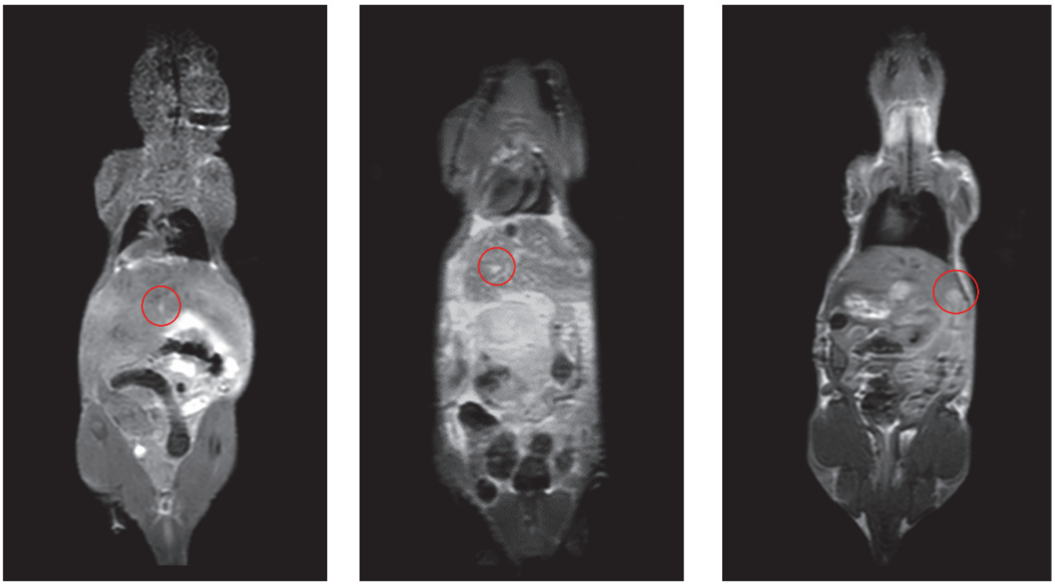

Supplementary Fig. 31 The mice with larger metastatic tumors. By MRI monitoring, such large metastases were excluded first. Data presented from individual mice sample. Source data are provided as a Source Data file.

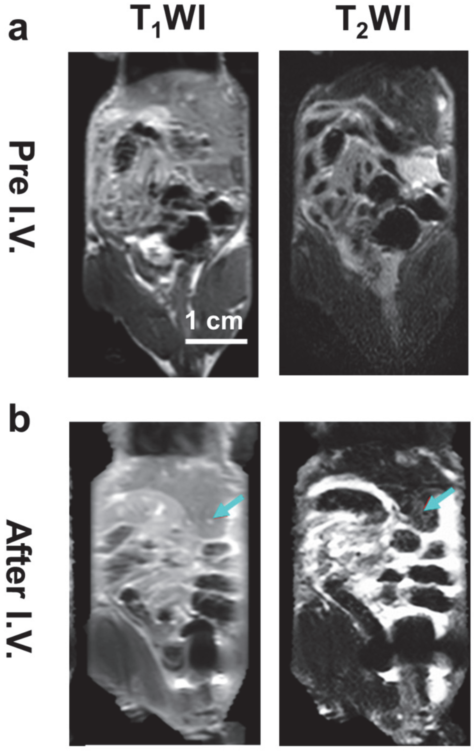

Supplementary Fig. 32 T_1 weighted image and T_2 weighted image of a liver metastatic model before (**a**) and after (**b**) i.v. injection of SSM-PEG. Representative data are shown from three mice samples. Source data are provided as a Source Data file.

Supplementary References

1. Lynch, J., *et al.* Gas-Bubble Effects on the Formation of Colloidal Iron Oxide Nanocrystals. *Journal of the American Chemical Society* **2011**, *133*, 12664-12674.

2. Shi, *et al.* Facile Synthesis of Folic Acid-Modified Iron Oxide Nanoparticles for Targeted MR Imaging in Pulmonary Tumor Xenografts. *Molecular imaging and biology: MIB: the official publication of the Academy of Molecular Imaging* **2016**, *18*, 569-578.
