# Supplementary material for: A pH-responsive T1-T2 dual-modal MRI contrast agent for cancer imaging
Source: Nat Commun. 2022 Dec 26;13:7948. doi: 10.1038/s41467-022-35655-x (PMC9792454; doi:10.1038/s41467-022-35655-x)
Supplement: Supplementary file 2 — Reporting Summary [file 41467_2022_35655_MOESM2_ESM.pdf]

nature portfolio

Corresponding author(s): Leilei Tian

Last updated by author(s): Dec 6, 2022

Reporting Summary

Nature Portfolio wishes to improve the reproducibility of the work that we publish. This form provides structure for consistency and transparency in reporting. For further information on Nature Portfolio policies, see our Editorial Policies and the Editorial Policy Checklist.

Statistics

For all statistical analyses, confirm that the following items are present in the figure legend, table legend, main text, or Methods section.

n/a	Confirmed	
☐	☒	The exact sample size (n) for each experimental group/condition, given as a discrete number and unit of measurement
☐	☒	A statement on whether measurements were taken from distinct samples or whether the same sample was measured repeatedly
☐	☒	The statistical test(s) used AND whether they are one- or two-sided<br>*Only common tests should be described solely by name; describe more complex techniques in the Methods section.*
☒	☐	A description of all covariates tested
☒	☐	A description of any assumptions or corrections, such as tests of normality and adjustment for multiple comparisons
☐	☒	A full description of the statistical parameters including central tendency (e.g. means) or other basic estimates (e.g. regression coefficient) AND variation (e.g. standard deviation) or associated estimates of uncertainty (e.g. confidence intervals)
☐	☒	For null hypothesis testing, the test statistic (e.g. F, t, r) with confidence intervals, effect sizes, degrees of freedom and P value noted<br>*Give P values as exact values whenever suitable.*
☒	☐	For Bayesian analysis, information on the choice of priors and Markov chain Monte Carlo settings
☒	☐	For hierarchical and complex designs, identification of the appropriate level for tests and full reporting of outcomes
☒	☐	Estimates of effect sizes (e.g. Cohen's d, Pearson's r), indicating how they were calculated

Our web collection on statistics for biologists contains articles on many of the points above.

Software and code

Policy information about availability of computer code

Data collection	Gatan Microscopy Suite 3; Zetasizer Nano ZS; UVProbe2.62; Gen5 2.09; Paravision 4; Image Lab 6.1; Bruker WinEPR 2.22
Data analysis	GraphPad Prism 7.00; Image J V 1.8.0; Matlab R2020a

For manuscripts utilizing custom algorithms or software that are central to the research but not yet described in published literature, software must be made available to editors and reviewers. We strongly encourage code deposition in a community repository (e.g. GitHub). See the Nature Portfolio guidelines for submitting code & software for further information.

Data

Policy information about availability of data

All manuscripts must include a data availability statement. This statement should provide the following information, where applicable:

- Accession codes, unique identifiers, or web links for publicly available datasets
- A description of any restrictions on data availability
- For clinical datasets or third party data, please ensure that the statement adheres to our policy

The data that support the findings of this study are available from the authors on reasonable request, see author contributions for specific data sets. Source data are provided with this paper.

Human research participants

Policy information about studies involving human research participants and Sex and Gender in Research.

Reporting on sex and gender	not applicable
Population characteristics	not applicable
Recruitment	not applicable
Ethics oversight	not applicable

Note that full information on the approval of the study protocol must also be provided in the manuscript.

Field-specific reporting

Please select the one below that is the best fit for your research. If you are not sure, read the appropriate sections before making your selection.

☒ Life sciences ☐ Behavioural & social sciences ☐ Ecological, evolutionary & environmental sciences

For a reference copy of the document with all sections, see nature.com/documents/nr-reporting-summary-flat.pdf

Life sciences study design

All studies must disclose on these points even when the disclosure is negative.

Sample size	All of the experimental data were obtained in triplicate unless otherwise mentioned and are presented as mean ± S.D. Statistical comparison by analysis of variance was performed at a significance level of $P < 0.05$ based on a Student's t-test.
Data exclusions	No data were excluded.
Replication	The cell viabilities were determined by MTT assay, and the results were presented as average ± SD (n = 3). Pharmacokinetic analyses were based on three mice per group. MRI analyses were based on three mice per group. All attempts at replication were successful.
Randomization	The allocation was random.
Blinding	The investigators were blinded to group allocation during data collection.

Reporting for specific materials, systems and methods

We require information from authors about some types of materials, experimental systems and methods used in many studies. Here, indicate whether each material, system or method listed is relevant to your study. If you are not sure if a list item applies to your research, read the appropriate section before selecting a response.

Materials & experimental systems

n/a	Involved in the study	
☒	☐	Antibodies
☐	☒	Eukaryotic cell lines
☒	☐	Palaeontology and archaeology
☐	☒	Animals and other organisms
☒	☐	Clinical data
☒	☐	Dual use research of concern

Methods

n/a	Involved in the study	
☒	☐	ChIP-seq
☒	☐	Flow cytometry
☒	☐	MRI-based neuroimaging

Eukaryotic cell lines

Policy information about cell lines and Sex and Gender in Research

Cell line source(s)	4T1 , 293T and U87 were purchased from Cell Bank of Chinese Academy of Sciences
Authentication	Cell lines authentication was performed by microscopic morphological observation and short tandem repeat DNA profiling
Mycoplasma contamination	The cell lines have been tested for mycoplasma contamination routinely
Commonly misidentified lines (See ICLAC register)	Not applicable

Animals and other research organisms

Policy information about studies involving animals; ARRIVE guidelines recommended for reporting animal research, and Sex and Gender in Research

Laboratory animals	BALB/c mice, 6-8 weeks;Nude mice, 6-8 weeks; Nude rat, 4 weeks.
Wild animals	No wild animals were used in the study.
Reporting on sex	Sex was not considered in this study.
Field-collected samples	No field collected samples were used in the study.
Ethics oversight	All animal experiments were in accord with Institutional Animal Use and Care Regulations, approved by the Laboratory Animal Ethics Committee of the Southern University of Science and Technology (No. SUSTC-JY2019068) and Shanghai General Hospital.

Note that full information on the approval of the study protocol must also be provided in the manuscript.
